# Association of mutations in the *Plasmodium falciparum* Kelch13 gene (*Pf3D7_1343700*) with parasite clearance rates after artemisinin-based treatments*—*a WWARN individual patient data meta-analysis

**DOI:** 10.1186/s12916-018-1207-3

**Published:** 2019-01-17

**Authors:** WWARN K13 Genotype-Phenotype Study Group, Chanaki Amaratunga, Voahangy Hanitriniaina Andrianaranjaka, Elizabeth Ashley, Delia Bethell, Anders Björkman, Craig A. Bonnington, Roland A. Cooper, Mehul Dhorda, Arjen Dondorp, Annette Erhart, Rick M. Fairhurst, Abul Faiz, Caterina Fanello, Mark M. Fukuda, Philippe Guérin, Rob Hooft van Huijsduijnen, Tran Tinh Hien, N. V. Hong, Ye Htut, Fang Huang, Georgina Humphreys, Mallika Imwong, Kalynn Kennon, Pharath Lim, Khin Lin, Chanthap Lon, Andreas Mårtensson, Mayfong Mayxay, Olugbenga Mokuolu, Ulrika Morris, Billy E. Ngasala, Alfred Amambua-Ngwa, Harald Noedl, François Nosten, Marie Onyamboko, Aung Pyae Phyo, Christopher V. Plowe, Sasithon Pukrittayakamee, Milijaona Randrianarivelojosia, Philip J. Rosenthal, David L. Saunders, Carol Hopkins Sibley, Frank Smithuis, Michele D. Spring, Paul Sondo, Sokunthea Sreng, Peter Starzengruber, Kasia Stepniewska, Seila Suon, Shannon Takala-Harrison, Kamala Thriemer, Nguyen Thuy-Nhien, Kyaw Myo Tun, Nicholas J. White, Charles Woodrow

**Affiliations:** Oxford, UK

## Abstract

**Background:**

*Plasmodium falciparum* infections with slow parasite clearance following artemisinin-based therapies are widespread in the Greater Mekong Subregion. A molecular marker of the slow clearance phenotype has been identified: single genetic changes within the propeller region of the Kelch13 protein (*pfk13*; *Pf3D7_1343700*). Global searches have identified almost 200 different non-synonymous mutant *pfk13* genotypes. Most mutations occur at low prevalence and have uncertain functional significance. To characterize the impact of different *pfk13* mutations on parasite clearance, we conducted an individual patient data meta-analysis of the associations between parasite clearance half-life (PC_1/2_) and *pfk13* genotype based on a large set of individual patient records from Asia and Africa.

**Methods:**

A systematic literature review following the PRISMA protocol was conducted to identify studies published between 2000 and 2017 which included frequent parasite counts and *pfk13* genotyping. Four databases (Ovid Medline, PubMed, Ovid Embase, and Web of Science Core Collection) were searched. Eighteen studies (15 from Asia, 2 from Africa, and one multicenter study with sites on both continents) met inclusion criteria and were shared. Associations between the log transformed PC_1/2_ values and *pfk13* genotype were assessed using multivariable regression models with random effects for study site.

**Results:**

Both the *pfk13* genotypes and the PC_1/2_ were available from 3250 (95%) patients (*n* = 3012 from Asia (93%), *n* = 238 from Africa (7%)). Among Asian isolates, all *pfk13* propeller region mutant alleles observed in five or more specific isolates were associated with a 1.5- to 2.7-fold longer geometric mean PC_1/2_ compared to the PC_1/2_ of wild type isolates (all *p* ≤ 0.002). In addition, mutant allele E252Q located in the *P. falciparum* region of *pfk13* was associated with 1.5-fold (95%CI 1.4–1.6) longer PC_1/2_. None of the isolates from four countries in Africa showed a significant difference between the PC_1/2_ of parasites with or without *pfk13* propeller region mutations.

Previously, the association of six *pfk13* propeller mutant alleles with delayed parasite clearance had been confirmed. This analysis demonstrates that 15 additional *pfk13* alleles are associated strongly with the slow-clearing phenotype in Southeast Asia.

**Conclusion:**

Pooled analysis associated 20 *pfk13* propeller region mutant alleles with the slow clearance phenotype, including 15 mutations not confirmed previously.

**Electronic supplementary material:**

The online version of this article (10.1186/s12916-018-1207-3) contains supplementary material, which is available to authorized users.

## Background

Artemisinin-based combination therapies (ACTs) have been the WHO recommended treatment for uncomplicated *Plasmodium falciparum* malaria since 2006. Studies conducted in 2006–2007 reported that *P*. *falciparum* parasites in northwest Cambodia had reduced in vivo susceptibility to artemisinins [1, 2]. This was manifest as delayed clearance of parasites from the blood of patients treated with artesunate monotherapy or ACTs [2]. In 2014, parasites selected in vitro for delayed response to artemisinins were found to have a  mutation in the *kelch13* gene (*pfk13*, *P*. *falciparum 3D7_1343700*) [3, 4], a locus consistent with genome association studies that had strongly associated a region of chromosome 13 with slow parasite clearance [5, 6]. Subsequent clinical studies in western Cambodia showed that delayed parasite clearance in clinical trials was associated with several non-synonymous mutations in *pfk13*. In particular, mutations in the distinctive propeller region of the Kelch 13 protein (codons 441–726) were associated with slow parasite clearance and subsequently with reduced artemisinin susceptibility in in vitro studies assessing susceptibility of the ring-stage parasites [3, 7, 8]. Recently, the structure of the propeller region was solved and made public by the Structural Genetics Consortium [9], making it possible to link particular genetic changes with their position in the molecule. A central role of *pfk13* propeller mutations in mediating ring-stage resistance was confirmed by demonstration that parasites engineered to contain the mutations showed ring-stage resistance to artemisinin whereas parasites with the wild type allele were sensitive [10].

As *pfk13* propeller sequences were determined in *P*. *falciparum* from the Greater Mekong Subregion (GMS), other Asian sites [6, 11–22], and Africa, Latin America, and Oceania [23–26]; almost 200 different non-synonymous mutations have been identified (see [27] and supplementary tables 6 and 7 in [28]).

The parasite clearance half-life (PC_1/2_), which measures the slope of the log-linear component of the parasite clearance curve, has become established as the best in vivo metric of *P*. *falciparum* artemisinin susceptibility [29–31]. Although sporadic parasite isolates with *pfk13* propeller mutations and occasional patients with slow parasite clearance have been identified in many parts of the malaria-endemic world, *pfk13* mutant parasites isolated from patients with the slow-clearing in vivo phenotype have been demonstrated only in a circumscribed region of Southeast Asia, initially in western Cambodia and currently extending to parts of Thailand, Vietnam, Lao PDR, Myanmar, and Yunnan Province, China. Within this region, there has been clear evidence of selection and spread of successful artemisinin-resistant parasite lineages [32]. As expected, artemisinin resistance has led to further selection of ACT partner drug resistance [33–35]. Outside the GMS, extensive molecular analyses have shown no evidence that parasites carrying *pfk13* propeller SNPs are under directional selection by artemisinins [23, 25, 27, 28, 36]. Taken together, available studies generally support the conclusion that certain *pfk13* propeller mutations mediate delayed parasite clearance following treatment with artemisinin derivatives in Southeast Asia, but that the *pfk13* propeller region mutants observed outside the GMS do not show an association with slow parasite clearance [37, 38]. Exceptions include recent molecular reports of independent emergence of parasites that carry *pfk13* propeller mutant alleles observed previously in the GMS in Guyana (C580Y) [39] and Rwanda (P574L and A675V) [40]. Accurate measurement of the parasite clearance phenotype requires frequent timed blood sampling [41]. This is often impractical and means that many surveys only collect information on the *pfk13* genotypes in a study area. It is therefore critical to utilize optimally those studies that do include both genotype and in vivo phenotype. The WHO has provided guidelines for associating *P*. *falciparum* parasite mutations with slow parasite clearance in vivo [42]. Currently, 6 *pfk13* propeller region mutant alleles have been validated in this way, 8 additional alleles have been described as “associated” with the phenotype, and 18 other alleles have been isolated in such low numbers that their phenotype could not be evaluated. It is also clear that some non-synonymous mutations in the *pfk13* propeller region (notably A578S) are not associated with artemisinin resistance. The low prevalence of *pfk13* propeller alleles outside the Greater Mekong Subregion has limited our ability to confirm the roles of additional genotypes in conferring the delayed clearance phenotype.

We have used meta-analysis of individual patient data from published and unpublished studies to compare standardized parasite clearance half-life estimates and *pfk13* propeller region genotypes. This analysis capitalized on the power of the large data set to allow assessment of the importance of a broader range of propeller region alleles in mediating slow parasite clearance in vivo*.*

## Methods

### Data acquisition

A systematic literature review following the PRISMA protocol [43] was conducted to identify studies published between 2000 and 2017 which included frequent parasite measurements and *pfk13* genotyping (last run on 31 January 2018). Four databases (Ovid Medline, PubMed, Ovid Embase, and Web of Science Core Collection) were searched. The search terms and conditions are available in Additional file 1.

At the abstract screening stage, we excluded studies that did not include treatment with artemisinin derivatives, *pfk13* genotyping or parasite measurements as well as review and correspondence articles. At full-text screening, we excluded additional studies that measured parasites less frequently than twice a day. Data were actively requested only from studies that reported parasites that carried *pfk13* non-synonymous mutations. However, we received two studies that found no parasites that carried these *pfk13* mutations and these were analyzed as part of the wild type data set.

Studies that identified non-synonymous mutations in *pfk13* were eligible for inclusion in this analysis if individual patient files also met the following criteria: (a) patients were treated with either an ACT or an artemisinin monotherapy; (b) parasitemia was measured in the first days of treatment at least every 12 h, until a negative count or at least until day 3, allowing the standardized calculation of the PC_1/2_ [29, 30]; (c) the dosing protocol was available; and (d) the weight of each individual patient was recorded. The published papers and data sources are listed in detail in Additional file 2: Table S1 [2, 11, 15, 16, 19, 44–51].

Individual study protocols were available for all trials included, either from the publication or as a metafile submitted with the primary data. Individual patient data from eligible studies were shared, collated, and standardized under the protocols of the WWARN data platform using previously described methodology [52]. Study reports generated from the formatted datasets were sent back to investigators for validation or clarification. Methodologies used in studies regarding parasitemia sampling and molecular analyses are presented in Additional file 2: Table S1.

### Statistical analysis

The statistical analysis plan [53] was developed before analysis. All non-synonymous mutations in the *pfk13* gene identified in the studies were included in the analysis. Isolates without reported mutations were assumed to be wild type in assessing relationships between parasite genotype and PC_1/2_. Isolates with a mixed genotype at any nucleotide within the *pfk13* coding region (wild type/mutation or two non-synonymous mutations) were excluded from the analysis.

The PC_1/2_ is defined as the time in hours needed for the parasite density to decline by 50% during the log-linear phase of parasite clearance. PC_1/2_ was calculated using the WWARN parasite clearance estimator tool [41]. The goodness of fit of parasite clearance models was evaluated for each individual patient parasitemia-time profile used to estimate the PC_1/2_.

Profiles that satisfied the following criteria (i.e., provided biologically or statistically plausible results) were included in the analysis: (a) standard deviations of residuals < 2, (b) number of data points used to fit the linear part of the curve > 2, (c) duration of lag phase < 12 h, (d) pseudo *R*^2^ statistics ≥ 0.8. Additionally, patients who withdrew or had a record of inadequate dosing were excluded. The log transformed half-life metric was modeled for all *pfk13* mutant alleles in all studies with information from individual patients on age, initial parasitemia, ACT treatment, and artesunate dose as covariates. The method by which the dose was calculated is documented in the statistical analysis plan. Random effects for study site were used to account for heterogeneity between studies. Residuals were examined for normality and for systematic deviations from the model.

The differences in PC_1/2_ between infections with *P*. *falciparum* parasites bearing a specific *pfk13* propeller mutant allele and those with wild type parasites were assessed by the Wald test. The fold change in geometric mean of PC_1/2_ of infections with *pfk13* mutant parasites compared to wild type isolates from the same sites; *x*PC_1/2_ was calculated as an exponent of the difference of the corresponding regression coefficients.

In order to determine a PC_1/2_ threshold value that defined slow parasite clearance, we divided Asian data into two populations: rapid clearing and slow clearing. The slow-clearing population was defined as all isolates with mutations associated with a significant increase in PC_1/2_ values in this analysis, while the fast-clearing population included all other isolates. The PC_1/2_ value that corresponds to the 95th percentile of the fast-clearing population (i.e., a value *x* such that the probability that PC_1/2_ > *x* is less than 0.05) was selected as the cutoff for infections with “slow clearing” parasites. Risk of bias in individual studies was assessed based on frequency of parasite counting, molecular methodology, and number of patients excluded because of missing data or unsatisfactory fit of the model for PC_1/2_ estimation (for details, see Additional file 1). Data from studies/sites that reported results very different from all of the others in the same region were included in the analysis, and a sensitivity analysis was conducted after excluding Tra Lang, Vietnam (study site ID 23; study ID 8), and Pyin Oo Lwin, Myanmar (study site ID 15; study ID 13).

## Results

### Literature search

The literature search identified 146 articles, with 9 satisfying our inclusion criteria and describing data from 14 studies (Additional file 1). Seven additional studies were contributed in response to the proposal for this study group on the WWARN website. Consequently, we requested individual patient data from 21 studies and received data from 18 studies.

### Description of the study sites

There were 16 studies from Asian sites that reported both PC_1/2_ estimates and *pfk13* propeller region genotypes from Myanmar, Thailand, Cambodia, Lao Peoples Democratic Republic (PDR), Vietnam, Bangladesh, and Yunnan province of China bordering Myanmar. Among these 3179 patients, 3012 had sufficient data for PC_1/2_ estimation and 2631 met the inclusion criteria for evaluation of the association of the *pfk13* genotype and the parasite clearance phenotype. Of the Asian isolates, nearly half (43%; 1142/2631) were from four study sites of the Shoklo Malaria Research Unit (SMRU) along the Western Thailand/Eastern Myanmar Border; these isolates are identified as a group called Thai Western Border.

**Fig. 1:**
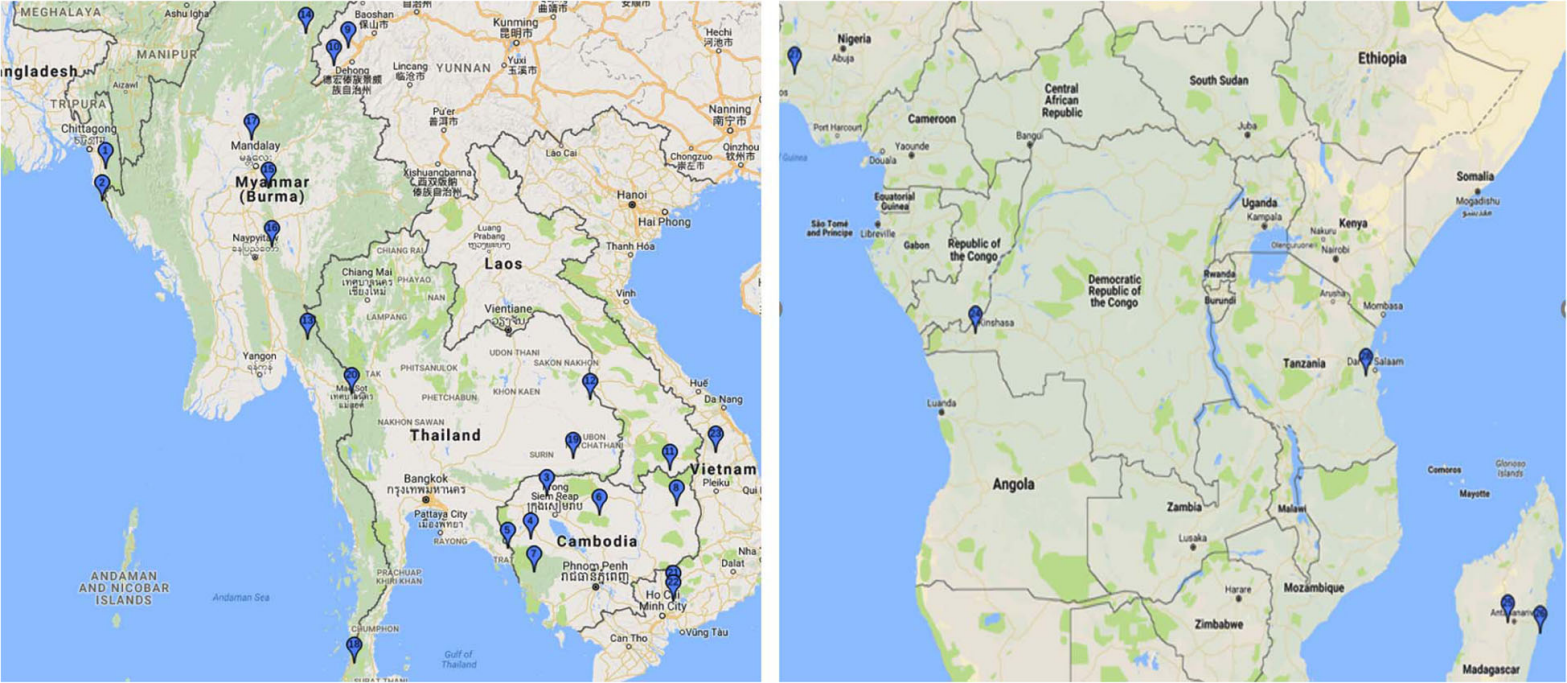
Study site locations. The numbers in the pins correspond to the study sites listed in Table 1

**Fig. 2:**
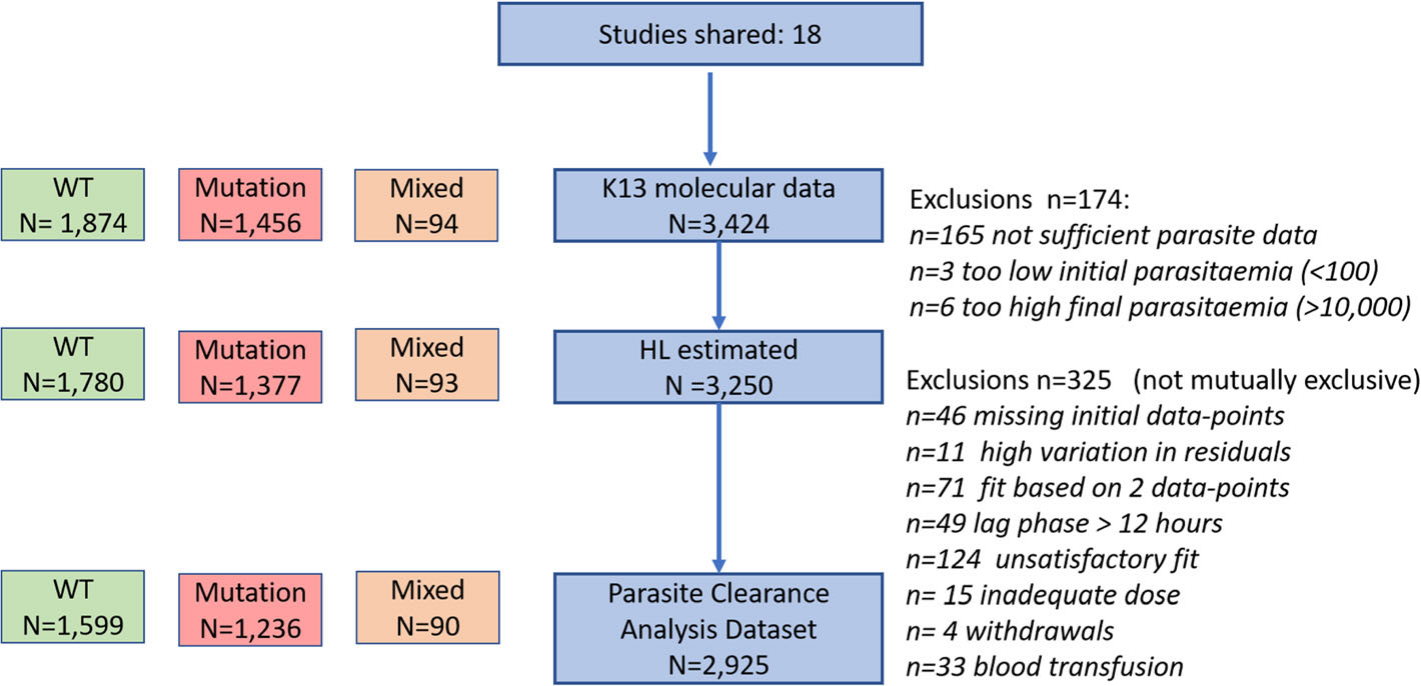
Study profile. Definitions for specific exclusions are listed at the right of the figure detailing the number of isolates included in each analysis. Unsatisfactory fit was defined as pseudo-*R*^2^ statistics < 0.8. Insufficient parasite data includes patients with too few observations to fit the model and patients with only daily counts

There were 238 isolates with genotype and phenotype information contributed from five sites in Africa; one site in Nigeria (*n* = 31), the Democratic Republic of Congo—DRC (*n* = 119), and Tanzania (*n* = 39); and two sites in Madagascar (*n* = 49). A total of 204 patients’ data met the inclusion criteria for evaluation of the association of the *pfk13* genotype and the parasite clearance phenotype (Table 1, Figs. 1 and 2).

**Table 1:** Summary of genotyping results by study site. Samples with mixed genotyping are excluded. Details of mutations are listed in Additional file 2: Table S2A (codons 1–440) and S2B (codons 441–726), and mixed genotypes are listed in Additional file 2: Table S3. Results are sorted by continent, country, site, and year of study

Study ID	Country	Site ID	Site	Year	Total *N* isolates	Non-synonymous changes between		
						Codon 1–440	Codon 441–726	
						*N*	*N*	% [95% CI]
Asia								
7	Bangladesh	1	Bandarban	2004–2005	21	2	0	0 [0–15]
16	Bangladesh	2	Ramu	2012	55	4	0	0 [0–7]
9	Cambodia	3	Anlong Veng	2012–2014	107	ND	103	96 [91–99]
2	Cambodia	4	Tasanh	2008–2009	46	ND	38	83 [69–91]
6	Cambodia	5	Pailin	2008–2009	36	ND	34	94 [82–98]
16	Cambodia	5	Pailin	2011–2012	87	0	80	92 [84–96]
10	Cambodia	6	Preah Vihear	2012–2013	65	ND	22	34 [24–46]
11	Cambodia	6	Preah Vihear	2011–2012	110	0	22	20 [14–28]
10	Cambodia	7	Pursat	2012–2013	107	ND	82	77 [68–84]
16	Cambodia	7	Pursat	2011–2012	104	0	77	74 [65–82]
10	Cambodia	8	Ratanakiri	2012–2013	66	ND	7	11 [5–20]
16	Cambodia	8	Ratanakiri	2011–2012	119	0	4	3 [1–8]
11	China	9	Tengchong	2012	12	ND	10	83 [55–95]
11	China	10	Yingjiang	2009–2012	110	ND	61	55 [46–64]
12	LPDR	11	Attapeu	2013–2014	18	0	4	22 [9–45]
14	LPDR	11	Attapeu	2011–2012	118	0	2	2 [0–6]
3	LPDR	12	Savannakhet	2010	33	0	0	0 [0–10]
15	Myanmar	13	Hpa Pun	2013	32	1	14	44 [28–61]
12	Myanmar	14	Myitkyina	2013–2014	43	0	21	49 [35–63]
13	Myanmar	15	Pyin Oo Lwin	2012–2014	31	0	15	48 [32–65]
16	Myanmar	16	Shwe Kyin	2011–2013	74	17	20	27 [18–38]
12	Myanmar	17	Thabeikkyin	2013–2014	71	1	12	17 [10–27]
16	Thailand	18	Ranong	2011–2012	19	0	13	68 [46–85]
16	Thailand	19	Srisaket	2011–2013	34	0	30	88 [73–95]
4	Thailand	20	TW border	2001	9	0	0	0 [0–30]
4	Thailand	20	TW border	2002	58	0	0	0 [0–6]
4	Thailand	20	TW border	2003	33	3	0	0 [0–10]
4	Thailand	20	TW border	2004–2006	28	0	0	0 [0–12]
4	Thailand	20	TW border	2007	26	2	1	4 [1–19]
4	Thailand	20	TW border	2008	341	30	57	17 [13–21]
1	Thailand	20	TW border	2008	5	1	1	20 [4–62]
4	Thailand	20	TW border	2009	227	38	47	21 [16–26]
4	Thailand	20	TW border	2010	138	20	46	33 [26–42]
4	Thailand	20	TW border	2011	113	17	66	58 [49–67]
16	Thailand	20	TW border	2011–2012	94	5	61	65 [55–74]
4	Thailand	20	TW border	2012	116	14	80	69 [60–77]
4	Thailand	20	TW border	2013	80	1	67	84 [74–90]
4	Thailand	20	TW border	2014	23	0	21	91 [73–98]
16	Viet Nam	21	Binh Phuoc	2011–2012	103	0	28	27 [20–36]
5	Viet Nam	22	Phuoc Long	2010–2011	92	0	34	37 [28–47]
8	Viet Nam	23	Tra Lang	2012	83	ND	67	81 [71–88]
Africa								
16	DRC	24	Kinshasa	2013	117	32	3	3 [1–7]
18	Madagascar	25	Ankazobe	2014	5	ND	0	0 [0–43]
18	Madagascar	26	Brickaville	2014	44	ND	4	9 [4–21]
16	Nigeria	27	Ilorin	2011–2012	36	14	0	0 [0–10]
17	Tanzania	28	Fukayosi	2012	41	ND	0	0 [0–10]

*DRC* Democratic Republic of Congo, *LPDR* Lao People’s Democratic Republic, *TW border* Thai Western border, *ND* not done

Figure 1 depicts the locations of the study sites. Additional file 3: Figure S2 summarizes the numbers of individual data sets that met all criteria for inclusion.

Patient characteristics and the numbers of patients treated with artesunate or artemisinin combination therapies are summarized in Table 2. The median age of patients in Asia was 21 years, and 77% were 12 years of age or older; African patients had a median age of 4.7 years, and only 6% were older than 11 years.

**Table 2:** Baseline characteristics and treatment administered. Only patients with genotyping and PC_1/2_ results are presented

	Asia		Africa	
	*N*	Median (range) or *N* (%)	*N*	*N* (%) or median (range)
Age (years)	2630	21 [0.1–70]	204	4.7 [0.7–29]
< 1 year		8 [0]		11 [5]
1–4 years		185 [7]		98 [48]
5–11 years		407 [15]		82 [40]
12+ years		2030 [77]		13 [6]
Parasitemia (microliter)	2631	97,214 [455–2,409,008]	204	53,507 [2240 - 605,329]
Temperature (C)	1658	38.2 [34.1–41.5]	204	37.6 [34.7–40.8]
Hemoglobin (g/dL)	770	13.1 [2.1–19.3]	26	10.8 [6.3–14.2]
Hematocrit (%)	1542	40 [12–55]	140	31 [21–44]
Artemisinin derivative: total 3 days dose (mg/kg)				
AL	0		80	9.3 [5.2–16.0]
AS	433	8.3 [1.0–49.7]	0	
AS + ACT^1^	1638	8.3 [0.5–28.9]	83	11.8 [4.7–15]
ASAQ	0		41	13.0 [8.6–16.7]
ASMQ	81	8.0 [3.8–16.1]	0	
DHAPIP	430	6.7 [3.0–17.8]	0	

^1^Artesunate was given in the first 3 days alone, followed by ACT

### Molecular analyses of the *pfk13* alleles

Samples were collected between 2001 and 2014. Among the 45 individual study sites, 33 included data from the entire *pfk13* gene (codons 1–726); 12 studies reported sequence of only the propeller region codons (441–726). A total of 202 and 1254 isolates with molecular information on mutants in codons 1–440 (*P*. *falciparum*-specific region) and codons 441–726 (the propeller region) were recorded respectively. The *pfk13* genotype of parasites from 3424 patients was determined; mutant alleles were identified in 1455 samples. The prevalence of mutant codons observed in each study is summarized in Table 1.

### *Plasmodium*-specific region (codons 1–440)

Data were available for the whole *pfk13* molecule in two African study sites: Kinshasa, DRC (study ID 13; site ID 24), and Ilorin, Nigeria (study ID 13; site ID 36). Ilorin had the higher prevalence of isolates with mutant codons in the *P*. *falciparum* domain (14/36, 39%), compared with Kinshasa (33/117, 28%). In 76% (37/49) of these isolates, the mutations were at codon 189; 35 carried the K189T mutation, 12 carried a synonymous K189K, and 2 carried a K189N codon (Additional file 2: Table S2A).

Complete *pfk13* DNA sequence was determined in 18 Asian study sites, including all of the studies from the Thai Western Border. Mutant codons in the 5′ region of the gene were rare in most Asian-derived samples. The K189T mutant codon was observed in samples from Bangladesh (study 7; site 1: *n* = 1/21, 5%), (study 16; site 2: *n* = 4/55, 7%) and two sites in Myanmar ((study 16; site 16: *n* = 3/74, 4%), study 12; site 17: *n* = 1/71, (1.4%)) (Additional file 2: Table S2A).

Many of the parasites from the Thai Western Border (site 20) shared an allele with a codon change at position 252 from glutamic acid to glutamine (E252Q). Parasites with this allele were observed in study 1 (*n* = 1/7, 14%), study 4 (*n* = 68/950, 7%), and study 16 (*n* = 14/116, 12%). Parasites with this genotype were also observed in Shwe Kyin in 2011–2013 (study 16, site 16), where they comprised 10/74 (14%) of the isolates tested (Additional file 2: Table S2A), and this allele has been observed near the Myanmar-China border, as well [14]. In the relatively conserved stem domain (350–440), mutants were observed only transiently in parasites from Shwe Kyin and the Thai Western Border.

### *pfk13* propeller region (codons 441–726)

The sequence of the *pfk13* propeller region was determined in all 45 data sets (Table 1, Additional file 2: Table S2B). In the African isolates, the only mutant codons identified in the propeller region were in 3/117 isolates from Kinshasa, each with a single mutation (codons S522C, A578S, or Q613L) and in 4/44 isolates from Brickaville, Madagascar, all with mutant codon A578S which is relatively common in Africa and has been reported in Thailand and Bangladesh [27]. The Asian data set was dominated by large numbers of patients from two regions, the Thai Western Border (*n* = 1291/3087 (42%)) and northwestern Cambodia (*n* = 847/3087 (27%)), with smaller numbers from Vietnam, Myanmar, the Myanmar-China Border, Bangladesh, and Lao PDR. The detailed data are summarized in Additional file 2: Table S2B. As has been published, the Thai Western Border, Thai, Cambodian, Laotian, and Vietnamese data sets contained numerous isolates with the *pfk13* C580Y allele [11–14, 18, 19, 21, 22, 28, 32, 34, 50, 54, 55]. In contrast, isolates with the Y493H and R539T mutant codons were common among Cambodian isolates, but uncommon in samples from Thailand with the exception of those from Srisaket, a province that borders Cambodia.

The F446I mutant predominated in data sets from Myanmar and the China/Myanmar border (*n* = 83/141, (59%) [14, 16, 27, 51]. The mutant codons in the propeller region identified among the African isolates, S522C, S613E, and the very common A578S “African” allele, were absent from the Asian data set.

### Association of *pfk13* mutations and parasite clearance half-life

Among 3329 patients with *pfk13* genotyping results that reported only a single mutation in the propeller region, PC_1/2_ values were estimated in 3156 (95%) patients. In 165 patients, PC_1/2_could not be estimated because the data were too sparse, the initial parasitemia was too low (< 100 parasites per microliter) (*n* = 3) or the last recorded parasitemia was too high (> 10,000 parasites per microliter) (*n* = 6). Overall, 90% (2834 of 3156) of PC_1/2_ estimates were classified as suitable for analysis (using criteria specified prospectively in the methods, and Fig. 2).

### Defining the groups of slow- and fast-clearing parasites

Two populations of parasites were identified among the Asian parasites: fast-clearing (log_e_ PC_1/2_ mean, 1.0 SD, 0.41) and slow-clearing including all isolates with mutations listed in Tables 3 and 7 (log_e_PC_1/2_ mean 1.9; SD 0.19). These groups had corresponding half-life geometric means of 2.7 h and 6.7 h, respectively. A PC_1/2_ equal to 5.5 h corresponds to the 95th percentile of the fast-clearing parasite population, and the probability of observing PC_1/2_ > 5.5 h in a single isolate coming from a sensitive population is equal to 0.043. On this basis, a median value of PC_1/2_ > 5.5 h was used to define parasites associated with slow clearance.

**Table 3:** Comparison of PC_1/2_ between patients with WT and a * pfk13* NS-mutation in Asia. Metric is the fold increase of PC_1/2_ in mutants vs. wild type. Only mutations with *n* ≥ 5 are shown. With only two exceptions (K189T and K438N), all patients that carried parasites with the indicated *pfk13* mutant codon showed a PC_1/2_ significantly different from those that carried WT parasites

			Comparison of PC_1/2_					
	*N* with	*N* with	Univariable			Multivariable^1^		
Codon	mutation	WT	*x*PC_1/2_	95%CI	*p* value	*x*PC_1/2_	95% CI	*p* value
K189T	8	148	1.1	0.8–1.4	0.729	1.1	0.8–1.4	0.644
E252Q	114	589	1.5	1.4–1.6	< 0.001	1.5	1.4–1.6	< 0.001
K438N	10	386	0.9	0.7–1.1	0.251	0.9	0.7–1.1	0.237
P441L	53	565	2.1	1.9–2.3	< 0.001	2.2	2.0–2.4	< 0.001
F446I	79	303	1.6	1.4–1.7	< 0.001	1.5	1.4–1.7	< 0.001
G449A	6	41	1.9	1.4–2.6	< 0.001	1.9	1.3–2.7	< 0.001
N458Y	34	520	2.5	2.2–2.8	< 0.001	2.5	2.2–2.8	< 0.001
M476I	8	481	2	1.6–2.5	< 0.001	2.0	1.5–2.5	< 0.001
A481V	5	410	1.8	1.3–2.4	< 0.001	1.6	1.2–2.2	0.002
Y493H	33	313	2.6	2.2–3.0	< 0.001	2.7	2.3–3.1	< 0.001
R515K	5	352	1.9	1.4–2.6	< 0.001	2.0	1.5–2.7	< 0.001
P527H/L*	23	422	1.7	1.5-2.0	< 0.001	1.7	1.5–2.0	< 0.001
N537I	8	231	1.7	1.3–2.2	< 0.001	1.8	1.4–2.3	< 0.001
G538V	24	558	1.8	1.6–2.1	< 0.001	1.9	1.6–2.2	< 0.001
R539T	76	369	2.1	1.9–2.4	< 0.001	2.1	1.9–2.4	< 0.001
I543T	92	156	1.9	1.7–2.3	< 0.001	2.1	1.8–2.4	< 0.001
I543T**	27	140	2.8	2.3–3.4	< 0.001	2.8	2.3–3.5	< 0.001
P553L	16	529	2.2	1.8–2.6	< 0.001	2.2	1.8–2.8	< 0.001
R561H	36	310	2.2	1.9–2.5	< 0.001	2.2	1.9–2.6	< 0.001
V568G	5	127	2.7	1.8–4.1	< 0.001	2.7	1.8–4.1	< 0.001
P574L	48	739	1.8	1.6–2.0	< 0.001	2.0	1.7–2.2	< 0.001
P574L***	35	723	1.8	1.6–2.1	< 0.001	1.8	1.6–2.1	< 0.001
C580Y	450	997	2.2	2.1–2.4	< 0.001	2.3	2.1–2.4	< 0.001
P667T****	5	50	2.2	1.6–2.9	< 0.001	2.1	1.6–2.9	< 0.001
A675V	49	501	2.2	1.9–2.4	< 0.001	2.2	2.0–2.4	< 0.001

^1^Adjusted for total artemisinin derivative dose in the first 3 days, partner drug, initial parasitemia and patient age

*20 P527H, one P527L, analyzed together, two P527H not analyzed since clearance data did not meet criteria

**With study ID 8, study site ID 23 (Tra Lang) removed

***With study ID 13, study site ID 15 (Pyin Oo Lwin) removed

****5 P667T, one P667T not analyzed since clearance data did not meet criteria

### African isolates

Summary statistics of PC_1/2_ estimates and their association with *pfk13* mutations are presented in Table 5 and Additional file 3: Figure S3A for wild type (median PC_1/2_ 2.2 h, range 0.7–6.3) and mutant K189T (median PC_1/2_ 2.1 h, range 0.8–7.1). Additional file 3: Figure S3B shows individual PC_1/2_ values for all other mutations observed in African isolates.

The PC_1/2_ values in wild type isolates from the five African sites were all well below the cutoff of 5.5 h, but varied significantly from one another (*p* = 0.004, Additional file 3: Figure S1). Compared to wild type isolates in Kinshasa, DRC, an area of high perennial transmission, the PC_1/2_ in wild type isolates was 1.36 times longer (95%CI 1.08–1.70; *p* = 0.008) in Brickaville, Madagascar, 1.46 times longer (95%CI 1.18–1.8; *p* = 0.001) in Ilorin, Nigeria; and not significantly different in Fukayosi, Tanzania (*p* = 0.117), or Ankazobe, Madagascar (*p* = 0.199) (Additional file 3: Figure S1).

**Fig. 3:**
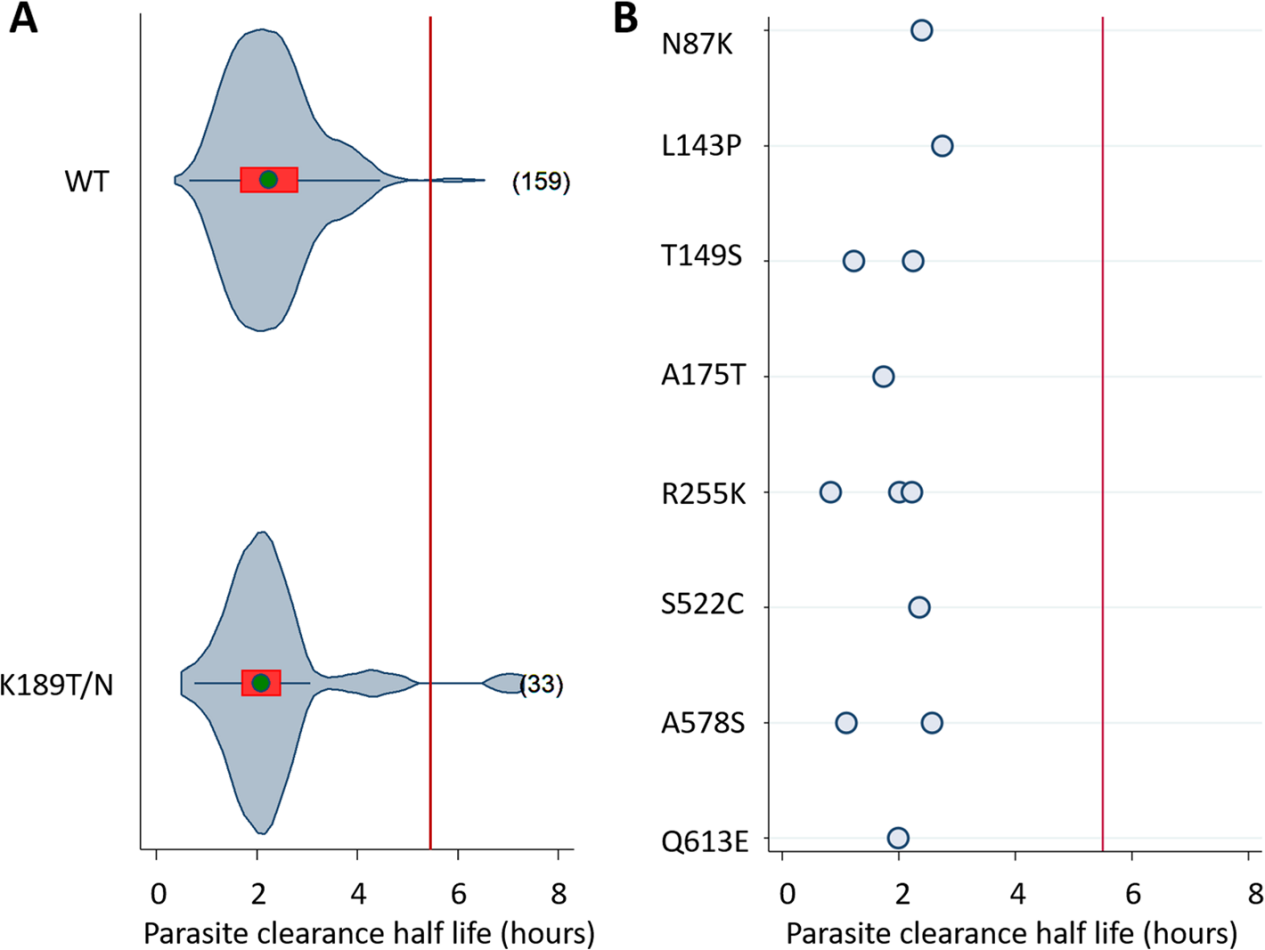
Distribution of parasite clearance half-life by individual wild type or *pfk13* mutant codons in isolates from Africa. Left panel (**a**) shows violin plots for wild type or mutant codons with five or more isolates. The number of individual isolates tested is at the right of each violin plot. The right panel (**b**) shows dot plots for mutant codons with < 5 isolates. The red line shows a half-life of 5.5 h. The median is shown as a green circle, the red bar corresponds to the interquartile range, and the curve represents kernel estimate of the density function

Most *pfk13* mutations were rare in the African data sets, being observed in fewer than four isolates (Figure 3). Among a total of seven isolates with a propeller mutation (S522C, A578S, or Q613L), four met the inclusion criteria for evaluation of the association of the *pfk13* genotype and the parasite clearance phenotype. All of the seven isolates had PC_1/2_ < 2.8 h and exhibited no increase in PC_1/2_ compared to infections with wild type parasites. In fact, the geometric mean of PC_1/2_ was lower in isolates with propeller mutations compared to wild type isolates at the same study sites (*p* = 0.491 and *p* = 0.096 when seven or four isolates were included). In this dataset, only the A578S allele of the propeller mutations observed in Africa was also observed in Asia, but in only three isolates. The median half-life for mutations anywhere in the *pfk13* gene ranged from 1.7 to 2.7 h (Additional file 3: Figure S3A and S3B).

Codon K189T/N was present in 33 isolates (31 were K189T and 2 were K189N), sufficient numbers to assess the overall parasite clearance; these infections had a median PC_1/2_ of 2.1 h (range 0.8–7.1) similar to that of infections with wild type parasites; 2.2 h (range 0.7–6.3) (Fig. 3a).

### Asian isolates

**Fig. 4:**
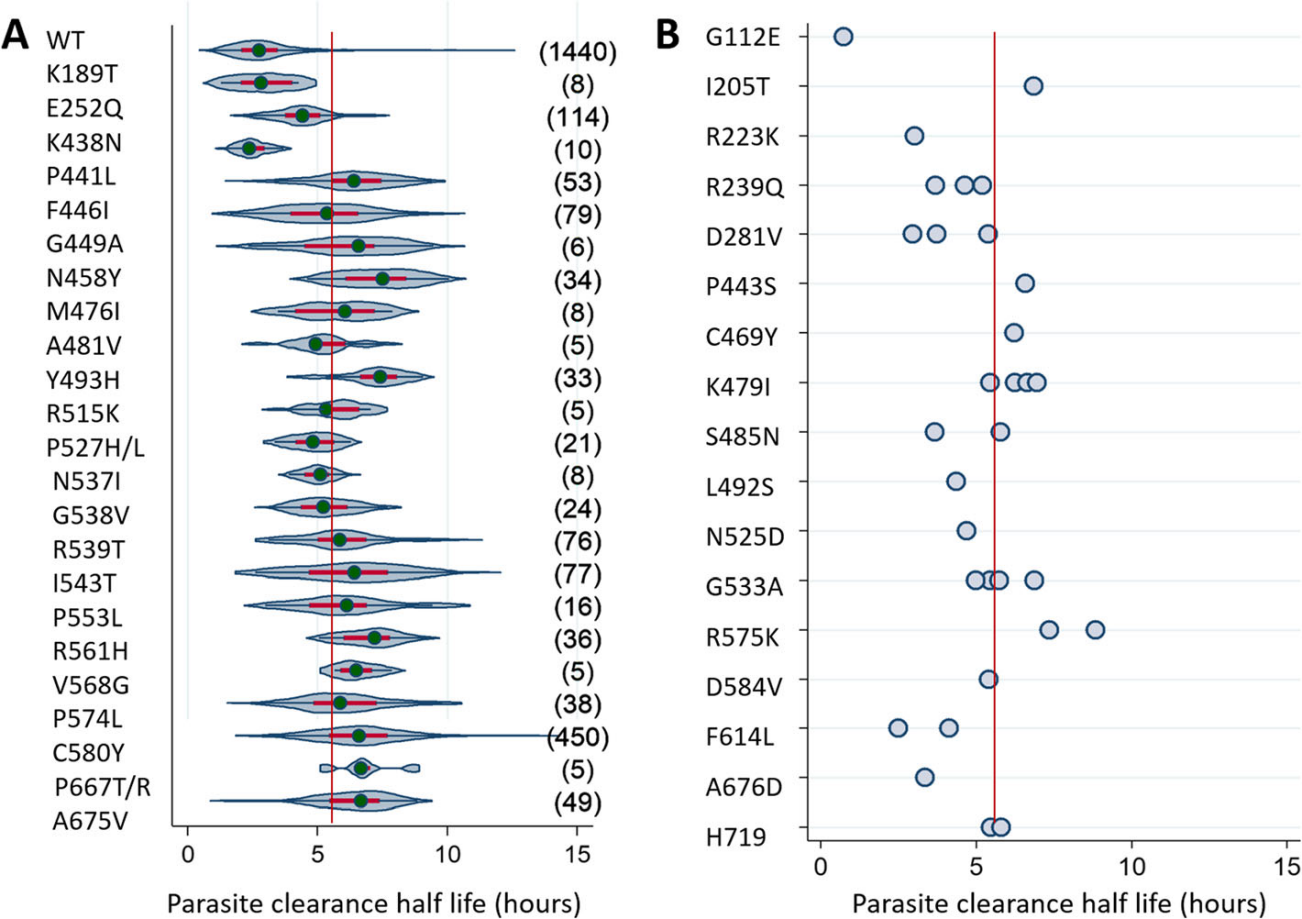
Distribution of parasite clearance half-life *by pfk13* mutant codons in isolates from Asia. Left panel (**a**) shows violin plots for mutant codons with five or more isolates. The number of individual isolates tested is at the right of each violin plot. The right panel (**b**) shows dot plots for mutant codons with < 5 isolates. The red line shows a half-life of 5.5 h. The median is shown as a green circle, the red bar corresponds to the interquartile range, and the curve represents kernel estimate of the density function. * 22 P527H and 1 P527L with PC1/2 = 5.8h; ** 6 P667T and 1 P667R without valid PC1/2 measurement, so not considered

Among Asian isolates, 1440 were wild type and 1190 carried a single *pfk13* mutation. The prevalence of isolates with a PC_1/2_ > 5.5 h and the median PC_1/2_ both varied depending on the *pfk13* mutation (Table 5, Fig. 4).

The 32 studies with infections caused by *P*. *falciparum* isolates that carried a non-synonymous change within codons 1–440 were analyzed, and all 45 studies with sequence information on the propeller regions were analyzed.

Variance in PC_1/2_ between genotypes was significantly larger than within genotypes (*F*_40, 2554_ = 48.43, *p* < 0.001, after adjusting for study site). All infections with *pfk13* mutants in the propeller region that were reported in five or more isolates were associated significantly with a geometric mean PC_1/2_ greater than that in infections with wild type parasites (*p* < 0.001 for all but codon A481V, in which *p* = 0.002). The fold increase in geometric mean of PC_1/2_ (*x*PC_1/2_), compared to the PC_1/2_ of wild type parasites at the same sites, ranged from 1.5 to 2.7 (Table 3). Parasites with mutations F446I, G449A, A481V, P527L, N537I, G538V, and P574L had a fold increase between 1.5 and 1.9, and all others were between 2.0 and 2.7. Neither isolates with the K189T mutation nor K438N showed any increase in PC_1/2_ compared with wild type parasites (*p* = 0.644 and 0.237, respectively). However, isolates with the E252Q mutation (which is also outside the propeller region) from both the Western Thai Border and Shwe Kyin, Myanmar, had a significant increase in PC_1/2_, 1.5-fold (*x*PC_1/2_ = 1.5; 95%CI 1.4–1.6) compared with wild type parasites (*p* < 0.001).

For infections caused by parasites that carried only wild type *pfk13*, the distribution of PC_1/2_ values varied significantly between locations (Additional file 3: Figure S2, *p* < 0.001); one site in Myanmar, Pyin Oo Lwin, and one in Vietnam, Tra Lang, had a median PC_1/2_ of wild type isolates that was above the cutoff of 5.5 h and above the PC_1/2_ observed for wild types in other sites (*p* < 0.001). At both sites, no significant differences were observed in PC_1/2_ between K13 wild type and K13 mutant isolates. The corresponding factor for change in PC_1/2_ in mutant isolates was estimated as 0.91 (0.74–1.12) *p* = 0.367 for Tra Lang, Vietnam, and 0.90 (0.73–1.10) *p* = 0.274 for Myanmar, Pyin Oo Lwin. Four mutant *pfk13* alleles were associated with significant differences in PC_1/2_ between different study sites: E252Q (*p* < 0.001), F446I (*p* = 0.005), M476I (*p* = 0.014), Y493H (*p* = 0.029) and C580Y (*p* < 0.001) (Table 4, Additional file 3: Figures S3-S7).

**Table 4:** Comparison of PC_1/2 for_ pfk13 NS mutations between study sites in Asia. Only mutations with isolates available from at least five patients from at least two sites are shown

Mutant codon	*N* sites	*N* patients	*p* value for comparison by site	
			Univariable	Multivariable^1^
K189T	3	8	0.324	0.278
E252Q	2	114	< 0.001	< 0.001
K438N	2	10	0.007	0.322
P441L	3	53	0.991	0.908
F446I	7	79	0.002	0.005
G449A	3	6	0.013	No data^2^
N458Y	2	34	0.583	0.249
M476I	3	8	< 0.001	0.014
A481V	2	5	0.664	No data^2^
Y493H	7	33	0.050	0.029
R515K	2	8	0.525	No data^2^
G538V	2	24	0.599	No data^2^
R539T	9	76	0.623	0.766
I543T	4	77	0.084	0.821
P553L	4	16	0.654	0.228
R561H	4	36	0.219	0.183
V568G	2	5	0.563	No data^2^
P574L	7	38	0.001	0.003
C580Y	13	450	< 0.001	< 0.001

^1^Adjusted for total artemisinin derivative dose in the first 3 days, partner drug, initial parasitemia and patient age

^2^Could not fit multivariable model due to small number of observations, overall or within sites

Between 2009 and 2014, the prevalence of the C580Y allele in parasite populations on the Thailand western border increased [18]. This change was accompanied by a progressive increase in the median PC_1/2_ of infections with *pfk13* C580Y mutants, from a median of 5.4 h in 2009 to 7.2 h in 2014 (Additional file 3: Figure S8). A linear trend of increase in the geometric mean of PC_1/2_ by 5.0% (95%CI 2.3–7.9) each year (*x*PC_1/2_ = 1.05; 95% CI 1.02–1.08) was observed (*p* < 0.001), and the linear trend was not affected by differences in patient treatment or artemisinin derivative dose.

### Influence of patient characteristics and antimalarial treatment drugs on PC_1/2_ in Asian isolates

Differences between PC_1/2_ values for parasites with the same *pfpk13* genotype were not significant when adjusted for patients’ initial parasitemia, artesunate dose, age, and study site. However, within the data set, patients whose parasite clearance was assessed had been treated with a range of artemisinin derivatives and doses and ACT partner drugs (Table 2). Groups with sufficient numbers of isolates (WT, F446I, Y493H, R539T, I543T, P553L, P574L, C580Y) were compared to assess the effects of the drugs administered on the half-life of parasite clearance. No differences in parasite clearance half-life among these different drug treatments were observed with one exception: the comparison between treatment with artesunate alone and artesunate/mefloquine in isolates with a WT genotype. Infections in patients treated initially with artesunate + mefloquine had a geometric mean PC_1/2_ 0.8 times (95%CI 0.7–0.9, *p* < 0.001) shorter than those treated with artesunate alone.

### Correspondence of PC_1/2_ > 5.5 h and day 3 parasitemia

Collecting frequent parasite counts in patients several times per day is not always feasible, and so recording the proportion of patients that remain parasitemic at day 3 after treatment has been proposed as a simple more practical warning signal for the possible slow clearance phenotype [29, 56]. To assess the correspondence between these two approaches, the proportion of patients with day 3 parasitemia in these data sets was compared with the proportion of isolates with a PC_1/2_ > 5.5 h (Table 5, Additional file 3: Figure S9). Use of the day 3 positivity test yielded an overall 11% (226/1975) false positive (FP) rate and 14% (107/772) false negative (FN) rate when compared to the use of the cutoff of PC_1/2_ > 5.5 h.

**Table 5:** Summary of PC_1/2_ by mutant codon and region showing proportion of isolates from a study site with PC_1/2_ > 5.5 h and proportion of Day 3 positive isolates. Mutant codons represented by fewer than 5 isolates are indicated in bold

	*N*	Patients with PC_1/2_ > 5.5 h (%)	PC_1/2_ Percentiles (hours)							Patients positive on day 3 (%)
			Median	25	75	5	95	Min	Max	
Asia										
WT	1440	5	2.7	2.1	3.4	1.4	5.3	0.7	12.4	9
**G112E**	**1**	**0**	**0.7**	**0.7**	**0.7**	**0.7**	**0.7**	**0.7**	**0.7**	**0**
K189 T	8	0	2.8	2.1	4.0	1.3	4.3	1.3	4.3	0
**I205T**	**1**	**100**	**6.8**	**6.8**	**6.8**	**6.8**	**6.8**	**6.8**	**6.8**	**100**
**R223K**	**1**	**0**	**3.0**	**3.0**	**3.0**	**3.0**	**3.0**	**3.0**	**3.0**	**0**
**R239Q**	**3**	**0**	**4.6**	**3.7**	**5.2**	**3.7**	**5.2**	**3.7**	**5.2**	**67**
E252Q	114	11	4.4	3.8	5.0	2.8	6.6	2.0	7.4	46
**D281V**	**3**	**0**	**3.7**	**2.9**	**5.4**	**2.9**	**5.4**	**2.9**	**5.4**	**33**
K438 N	10	0	2.4	2.2	2.9	1.4	3.7	1.4	3.7	30
P441L	53	75	6.4	5.6	7.4	3.5	8.9	2.0	9.4	77
**P443S**	**1**	**100**	**6.6**	**6.6**	**6.6**	**6.6**	**6.6**	**6.6**	**6.6**	**100**
F446I	79	47	5.4	4.0	6.5	2.3	8.6	1.6	10.0	32
G449A	6	67	6.6	4.5	7.1	2.3	9.5	2.3	9.5	67
N458Y	34	88	7.5	6.1	8.4	5.0	9.8	5.6	10.1	91
**C469Y**	**1**	**100**	**6.2**	**6.2**	**6.2**	**6.2**	**6.2**	**6.2**	**6.2**	**100**
M476I	8	63	6.1	4.2	7.1	3.5	7.9	3.5	7.9	71
**K479I**	**4**	**75**	**6.4**	**5.8**	**6.8**	**5.4**	**7.0**	**5.4**	**7.0**	**100**
A481V	5	40	4.9	4.7	6.0	2.7	7.6	2.7	7.6	40
**S485N**	**2**	**50**	**4.7**	**3.7**	**5.8**	**3.7**	**5.8**	**3.7**	**5.8**	**50**
**L492S**	**1**	**0**	**4.3**	**4.3**	**4.3**	**4.3**	**4.3**	**4.3**	**4.3**	**0**
Y493H	33	82	7.4	6.7	8.0	4.3	9.0	4.3	9.1	84
R515K	5	40	5.3	5.3	6.6	3.5	7.1	3.5	7.1	80
**N525D**	**1**	**0**	**4.7**	**4.7**	**4.7**	**4.7**	**4.7**	**4.7**	**4.7**	**0**
P527H/L*	21	29	4.8	4.2	5.6	3.4	6.0	3.3	6.3	67
**G533A**	**4**	**50**	**5.6**	**5.2**	**6.3**	**5.0**	**6.9**	**5.0**	**6.9**	**100**
N537I	8	13	5.1	4.5	5.4	3.9	6.3	3.9	6.3	63
G538 V	24	46	5.2	4.4	6.1	4.2	7.2	3.1	7.7	48
R539T	76	61	5.8	5.0	6.8	3.3	9.3	3.1	10.9	56
I543T	77	70	6.4	4.7	7.7	3.0	9.2	2.6	11.3	52
P553L	16	69	6.1	4.7	6.8	3.0	10.1	3.0	10.1	63
R561H	36	97	7.2	6.1	7.7	5.5	9.1	5.0	9.2	92
V568G	5	100	6.5	5.9	7.0	5.7	7.9	5.7	7.9	100
P574L	38	61	5.9	4.9	7.2	3.6	9.6	2.3	9.8	47
**R575K**	**2**	**100**	**8.1**	**7.3**	**8.8**	**7.3**	**8.8**	**7.3**	**8.8**	**100**
C580Y	450	74	6.6	5.5	7.6	4.1	9.3	2.3	13.9	75
**D584V**	**1**	**0**	**5.4**	**5.4**	**5.4**	**5.4**	**5.4**	**5.4**	**5.4**	**100**
**F614L**	**2**	**0**	**3.3**	**2.5**	**4.1**	**2.5**	**4.1**	**2.5**	**4.1**	**50**
P667T**	5	80	6.7	6.5	7.0	5.3	8.7	5.3	8.7	100
A675V	49	76	6.7	5.5	7.3	3.4	8.4	1.4	8.8	**80**
**A676D**	**1**	**0**	**3.4**	**3.4**	**3.4**	**3.4**	**3.4**	**3.4**	**3.4**	**0**
**H719N**	**2**	**50**	**5.6**	**5.5**	**5.8**	**5.5**	**5.8**	**5.5**	**5.8**	**50**
Africa										
WT	159	1	2.2	1.7	2.8	1.2	4.0	0.7	6.3	1
N87K	**1**	**0**	**2.4**	**2.4**	**2.4**	**2.4**	**2.4**	**2.4**	**2.4**	**0**
L143P	**1**	**0**	**2.7**	**2.7**	**2.7**	**2.7**	**2.7**	**2.7**	**2.7**	**0**
T149S	**2**	**0**	**1.7**	**1.2**	**2.2**	**1.2**	**2.2**	**1.2**	**2.2**	**0**
A175T	**1**	**0**	**1.7**	**1.7**	**1.7**	**1.7**	**1.7**	**1.7**	**1.7**	**0**
K189 T/N	33	3	2.1	1.7	2.5	0.9	4.6	0.8	7.1	0
R255K	**3**	**0**	**2.0**	**0.8**	**2.2**	**0.8**	**2.2**	**0.8**	**2.2**	**0**
S522C	**1**	**0**	**2.4**	**2.4**	**2.4**	**2.4**	**2.4**	**2.4**	**2.4**	**0**
A578S	**2**	**0**	**1.8**	**1.1**	**2.6**	**1.1**	**2.6**	**1.1**	**2.6**	**0**
Q613E	**1**	**0**	**2.0**	**2.0**	**2.0**	**2.0**	**2.0**	**2.0**	**2.0**	**0**

*20 P527H, one P527L, analyzed together; two P527H not analyzed since clearance data did not meet criteria

**5 P667T; one P667T not analyzed since clearance data did not meet criteria

However, since the initial parasitemia is a major determinant of time to clear microscopy detectable parasites after treatment, the proportion of FP and FN varies across a range of initial parasitemia values with 51% FN for parasite numbers less than 10,000/μL and 29% FP for parasite numbers greater than 300,000/μL (Table 6).

**Table 6:** Utility of day 3 parasite positivity for detection of isolates with long PC_1/2_ (> 5.5 h). False positive and false negative outcomes are estimated over a range of initial parasitemia values

Parasitemia (/μL)	*N*	False positive %	False negative %
< 10,000	127	0	51
10,000–50,000	491	3	26
50,000–100,000	804	5	8
100,000–300,000	887	14	3
≥ 300,000	438	29	0
All	2747	11	14

### Association of additional *pfk13* mutant alleles with slow parasite clearance

The current pooled analysis has allowed definition of additional *pfk13* genotypes that are associated strongly with slow parasite clearance (Tables 3 and 7). Six *pfk13* genotypes have already been associated with slow clearance by WHO, and 11 alleles were listed as candidates [57]. These have all now been demonstrated to be strongly associated with slow clearance (*p* < 0.001 except allele A481V, *p* = 0.002). In addition, 6 alleles that previously had been observed infrequently were present in high enough numbers in the analysis data set to allow assessment of the associated PC_1/2_; these alleles also showed a strong association with prolonged parasite clearance. All of the associated mutant alleles had single changes in the *pfk13* propeller region, with one exception, allele E252Q, located in the N-terminal *Plasmodium*-specific region of the protein. Two alleles with a smaller effect on parasite clearance, E252Q and F446I, could now be assessed with confidence since a larger sample size was available (*n* = 703 and *n* = 382, respectively). Parasites with the *pfk13* E252Q mutation had a median PC_1/2_ equal to 4.4 h and 11% had a PC_1/2_ > 5.5 h, those that carried a F446I mutation had a median PC_1/2_ of 5.4 h and a PC_1/2_ > 5.5 h was recorded in 47% of the patients.

**Table 7:** List of new *pfk13* mutant alleles strongly associated with slow parasite clearance

WHO designations		This paper					
Codon	Status	Codon	*N* total isolates	# sites	*N* mutant	% Prevalence: Median (Range)^1^	*x*PC_1/2_ (95% CI)^2^
*pfk13* mutant alleles associated with slow parasite clearance							
N458Y	Validated	N458Y	1118	2	38	3 (2–4)	2.5 (2.2–2.9)
Y493H	Validated	Y493H	638	8	41	4 (2–19)	2.7 (2.3–3.1)
R539T	Validated	R539T	767	10	80	7 (2–53)	2.1 (1.9–2.4)
I543T	Validated	I543T	323	4	94	13 (4–81)	2.1 (1.8–2.4)
		I543T*	240	3	27	10 (4–16)	2.8 (2.3–3.5)
R561H	Validated	R561H	1083	4	42	4 (3–6)	2.2 (1.9–2.6)
C580Y	Validated	C580Y	2343	14	536	21 (2–75)	2.3 (2.1–2.4)
*pfk13* mutant alleles newly associated with slow clearance phenotype							
E252Q	Not assoc	E252Q	1236	2	124	13 (10–16)	1.5 (1.4–1.6)
P441L	Candidate	P441L	1248	3	67	8 (5–10)	2.2 (2.0–2.4)
F446I	Candidate	F446I	764	7	98	31 (1–67)	1.5(1.4–1.7)
G449A	Candidate	G449A	88	3	7	15 (3–25)	1.9 (1.3–2.7)
M476I	Low prev	M476I	886	3	10	3 (1–3)	2.0 (1.5–2.5)
A481V	Low prev	A481V	852	3	10	8 (1–9)	1.6 (1.2–2.2)
R515K	Low prev	R515K	573	1	6	1 (1–1)	2.0 (1.5–2.7)
P527H	Low prev	P527H/L**	711	1	23	3 (3–3)	1.7 (1.5–2.0)
N537I/D	Low prev	N537I	656	2	10	3 (1–4)	1.8 (1.4–2.4)
G538V	Candidate	G538V	1163	2	27	2 (2–2)	1.9 (1.6–2.2)
P553L	Candidate	P553L	1112	4	18	6 (1–12)	2.2 (1.8–2.8)
V568G	Candidate	V568G	195	2	6	3 (2–4)	2.7 (1.8–4.1)
P574L	Candidate	P574L	1203	8	48	6 (2–50)	2.0 (1.7–2.2)
		P574L***	1177	7	35	5 (2–17)	1.8 (1.6–2.1)
P667T	Low prev	P667T	346	1	7	2 (2–2)	2.1 (1.6–2.9)****
A675V	Candidate	A675V	1114	1	53	5 (5–5)	2.2 (2.0–2.4)

^1^Prevalence is calculated per study site, combining data from all studies in that location, restricted to range of years when the mutation was observed

^2^Increase in PC_1/2_ compared to wild type; all *p* values for comparison with WT < 0.001. All have a half-life between 1.5 and 2.7 fold greater than the wild type. WHO designations can be found in [57]

*With study ID 8, study site ID 23 (Tra Lang) removed

**20 P527H, one P527L, analyzed together, two P527H not analyzed since clearance data did not meet criteria

***With study ID 13, study site ID 15 (Pyin Oo Lwin) removed

****6 P667T, 1 P667R not analyzed since clearance data did not meet criteria

### Risk of bias

The risk of bias in individual studies was considered low for majority of studies (Additional file 1).

Sensitivity analysis performed after exclusion of the only study (study ID 8, site ID 23) with moderate risk of bias due to genotyping methods shows the same results as analysis on the full dataset (Table 3). Only the mutant codon I543T was reported at this study/site. Additionally, one study site (study ID 13, site ID 15) presented significantly different PC_1/2_ results from other sites for parasites with no *pfk13* mutant codons (Additional file 3: Figure S1). The mutant codon P574L predominated in that site. We tested the sensitivity of the association of codon P574L with slow clearance by including and excluding the site ID 15 data (Table 3 and Table 7). The association of the P574L codon with slow parasite clearance was highly significant in both analyses (*p* ≤ 0.001).

Data from three apparently relevant published studies were not shared [3, 22, 38]. However, the association of particular mutations with slow clearing parasites coincides with our analysis. In Southeast Asia, alleles Y493H, 539T, I543T, P553L, V568G, and C580Y in Thuy-Nhien et al. [22] and C580Y, Y493H, and R539T in Ariey et al. [3] were associated with slow clearance, and in Mali, the 9 *pfk13* mutant parasites identified by Ouattara et al. were not associated with slow clearance [38].

## Discussion

This is the largest evaluation of the association of different *pfk13* mutations with slow parasite clearance following antimalarial treatment with the artemisinin derivatives. Our study confirms that many, but not all (notably A578S), mutations in the propeller region of the *pfk13* gene are associated with slow parasite clearance. It also shows that a mutation outside the propeller region, E252Q which had been proposed to confer artemisinin resistance, is strongly associated with slow parasite clearance. This is also consistent with the increased prevalence for several years on the northwest Thailand-Myanmar border of parasites that carried the E252Q mutation, presumably as a result of selection, before the ascendance of the C580Y genotype conferring a more extreme phenotype [18].

Several factors affect the rates of parasite clearance following administration of artemisinin derivatives and would have contributed to the inter-individual and between-site differences observed in this study. In infections with *pfk13* wild type parasites, treatment with an artemisinin derivative results in accelerated ring-form clearance, and this is reflected in a steep slope of the parasitemia-time profile and a derived PC_1/2_ which is usually well below 5.5 h. Background immunity, or its surrogate, age has a significant additional effect, further accelerating parasite clearance. Thus, immunity as reflected by antibody concentrations can have a relatively small but significant effect [58]. Another important contributor to the half-life is the stage of parasite development at the initial presentation of the patient. This variable, too, could disproportionately affect clearance of *pfk13* mutant parasites compared to wild type infections [59] but should only affect inter-individual and not inter-site differences. In artemisinin combination treatments, the partner drug also makes a small contribution to the rate of parasite clearance, which in this study, was significant only for the mefloquine combination, confirming previous findings [60]. It is important to note that despite modest site-specific differences, the clearance half-life derived for each *pf13* propeller alleles in the pooled data set was at least 1.5-fold higher than the half-life of the wild type parasites, and for 14 of the 20 propeller mutant alleles, the ratio of mutant to wild type PC_1/2_ was between 2.0 and 2.7.

The prominent role of *pfk13* propeller mutants in the selective response to artemisinin drug pressure has been recently demonstrated by the rapid increase in parasites that carry a genotype with a particular version of the C580Y allele. These parasites originated in northwestern Cambodian and Thai foci and spread recently to new areas of Cambodia and Vietnam [22, 32]. On a more local scale, in this study, we found that the median PC_1/2_ of parasites that already carried a C580Y allele of *pfk13* increased from 5.4 h in 2009 to 7.2 h in 2014 in the western border region of Thailand. More widely, among the 14 *pfk13* propeller mutants newly associated with slow clearance, 4 of the 14 mutant alleles associated with slow clearance were present in at least 8% of the isolates identified in the corresponding parasite population (range 8–31%). Ten of the newly identified *pfk13* mutant alleles were present at low prevalence (range 1–6%) in the sites where they have been reported and even among the validated alleles, prevalence was below 10% for four of the six alleles. Because the pooled data set contains isolates collected from many different sites and over a time span of 14 years, no trends in overall prevalence can be inferred in this analysis. However, the value of following temporal changes in the prevalence of propeller mutants in local sites is clear, as has been demonstrated for parasites that carry the C580Y allele in Thailand, Cambodia, and Vietnam [22, 32].

Detailed temporal studies *pfk13* mutant allele prevalence both in the GMS and in other worldwide sites represent a powerful tool for assessing whether a particular *pfk13* mutant population is increasing, a signal that further studies of the parasite phenotype may be warranted in that location. Temporal studies will be particularly important for the seven newly identified propeller mutant alleles in our data set that were observed at prevalences of 4% or lower.

In contrast, the *pfk13* mutant parasites in all five African sites remained at very low prevalence, generally below 3%, and no evidence of slow-clearing parasites or selection for mutant parasites has been identified. The low prevalence of *pfk13* mutant parasites in the African studies in our data set depends on relatively small numbers of parasites (e.g., 31, 26, 29). However, the generality of this observation is supported by extensive studies in many sites in Africa including some assessments of parasite clearance in vivo [37, 38, 61–63], protection against parasite exposure to artemisinins of cultured parasites in vitro [10, 62, 64], and widespread molecular surveillance of *pfk13* propeller region sequence ([23–26, 55, 65–68] and see the WWARN K13 surveyor and WHO drug resistance threats map for details). These many reports also support the conclusions of our study: in African sites, *pfk13* propeller mutants are diverse but rare, with no evidence for selection even where artemisinin-based antimalarials have been intensively used. Moreover, the *pfk13* propeller mutant alleles commonly observed in Asia have rarely been observed in Africa [40, 69]. In addition, only two African sites report a somewhat higher prevalence of parasites with *pfk13* propeller mutations, 14% (18/130) in Mali [23] and an increase from 3% (1/31) in 2007 to 27% (7/26) in 2014 in Grande Comoros [70] and 17% (5/29) mutants in individuals from the neighboring island of Mayotte [71].

The *pfk13* gene has been identified as essential to *P*. *falciparum* in its blood stream stage [72], and it is likely that parasites dependent upon a mutant K13 protein would incur a considerable fitness disadvantage [64]. In this situation, parasite populations subjected to intensive use of artemisinin-based antimalarials would be expected to be under intense selection for *pfk13* mutant parasites that carry additional genetic changes that could compensate for the fitness cost as has been observed in other parasite populations under drug selection [73]. There is increasing evidence that such adaptive responses have evolved in Southeast Asian *P. falciparum* populations [74]. For example, intensive use of the ACT dihydroartemisinin-piperaquine in Cambodia and Thailand has apparently exerted further selection on the parasites that carry the *pfk13* C580Y “spreading” genotype, selecting parasites that also carry an increased copy number of *plasmepsin 2–3* that confers resistance to the piperaquine partner drug [33–35]. In addition to selection and spread of *pfk13* mutant parasites, independent populations that carry new versions of the common C580Y or one of many novel *pfk13* alleles have emerged in many other sites within the GMS region [12–15, 21, 22, 28, 54].

Even before the *pfk13* gene was identified as a potential molecular marker of reduced artemisinin response, genome-wide association studies identified several regions of the *P. falciparum* genome associated with slow parasite clearance on chromosomes 10 and 14, in addition to chromosome 13 where the *pfk13* gene is located [5, 6, 75]. Whole genome analyses have also identified genetic changes associated both with *pfk13* propeller mutants and with slow clearance in independent, low diversity populations in Cambodia [76, 77] and in Western Thailand [78]. Parasites that carry these additional genetic changes are present in many populations in the western regions of Southeast Asia [21, 28, 79]. Moreover, the importance of other genetic changes for the expression of the slow clearance phenotype has been observed in vitro. Introduction of a *pfk13* mutant allele into a cloned, fast-clearing wild type parasite of recent Cambodian origin conferred more protection against dihydroartemisinin in vitro than when the same allele was expressed in a wild type parasite line of African origin [10]. These observations support the interpretation that the independent emergence of parasite populations with new *pfk13* alleles is facilitated in Southeast Asia by the presence of local parasite populations with genomes already adapted to support *pk13* mutant parasites when they arise. Exposure to artemisinins could then quite rapidly select emergence and selection of a local population of *pfk13* mutant parasites.

These specific adaptive genomic changes that are common in Southeast Asian parasites have not been observed in African isolates, but comprehensive analyses have not yet been undertaken [28]. The slow clearance phenotype is extremely complex, dependent on changes in the overall stress response to artemisinins (see [80, 81] for comprehensive reviews) and there may be many other combinations of adaptive genomic changes that could support the fitness of *pfk13* mutant parasites. In addition, it is clear that genetic changes other than *pfk13* mutations can confer diminished response to artemisinins in vitro and in vivo [82–85] and surveillance plans will need to remain open to the possibility of these alternative genetic strategies.

This meta-analysis still depends on detailed multiple quantitative assessments of the parasite count, not an approach that is likely to be feasible for routine surveillance in many study sites. This study highlights the limitations in sensitivity and specificity of the standard assessment of parasite clearance on day 3 as a phenotypic signal of delayed clearance. There is no question that simpler methods for assessment of parasite responses to artemisinins will be needed in all endemic areas, with particular attention to areas where malaria transmission is low and drug pressure will be most likely to select resistance.

Our large data set allowed identification of significant association of *pfk13* parasite genotype and PC_1/2_ phenotype even when the sample size of parasites with a particular allele was very small. For example, four different mutant alleles were observed in fewer than eight patient isolates. Even in these cases, the analysis allowed a strong genotype-phenotype association to be identified. Among these rare mutant alleles, the ratio of mutant to wild type PC_1/2_ ranged from 1.9 to 2.7, ten of the mutant alleles are present in more than 10% of the parasites in the tested isolates, and all but four were observed in more than one study site. This robust outcome demonstrates that analyses of the PC_1/2_ in patients can allow identification of mutant *pfk13* alleles associated with slow-clearing parasites even when malaria transmission is low and few patients can be assessed. This suggests that some researchers in areas outside the GMS may be able to assess the PC_1/2_ phenotype in vivo [11, 37, 38, 61] or parasite responses to artemisinins in vitro [10, 62, 85–87] as a screen for artemisinin response as some groups have already done.

Finally, 18 data sets were included in this meta-analysis of individual patient data sets, including 4 trials that had not yet been published. We believe that this demonstrates that the engagement of research groups to share individual patient data can provide a unique opportunity for advancement of public health. This approach can combine and facilitate collaborative analysis of critical data sets, defining outcomes that cannot be achieved by only extracting outcomes from multiple single publications. Such meta-analyses can provide evidence for policy makers, while not precluding subsequent individual study publications.

There are limitations of this meta-analysis. Notably, the uneven representation of isolates derived from different sites of origin between Africa and Asia and within Southeast Asia and the imbalance of longitudinal data among the sites. The extensive long-term record of clinical outcomes of artemisinin treatment from patients on the Thai Western Border contributed many more isolates to the available data set than any other site (1291/3087, 42%). However, this rich sample set is itself diverse, contributing information on 31 of the 44 mutant alleles observed in the Asian data set. Moreover, the patients studied were not from a very limited area, but came from sites situated over an extended region of the northwest Thai/eastern Myanmar border. The diversity of propeller mutant genotypes supports the assumption that this large number of isolates from the Thai Western Border is representative of the Eastern part of the whole region that includes Thailand, Cambodia, Lao PDR, and Vietnam.

## Conclusions

The slow clearance phenotype that has evolved in *P. falciparum* parasite populations in response to artemisinin challenge is complex and can involve at least one non-synonymous change in the *P. falciparum-*specific region of the *pfk13* gene, but most frequently, specific single mutations in the propeller region. These different propeller mutations may also compromise parasite fitness, so genomic studies that further define the other genomic changes in parasite populations that support *pfk13* mutants to overcome fitness deficits will be needed.

Despite this complexity of the phenotype, the appearance and selection of mutations in the *pfk13* propeller region are valuable markers for surveillance of diminished artemisinin responsiveness in parasite populations. This meta-analysis demonstrates that 14 *pfk13* propeller mutants, in addition to the 6 previously validated, are associated with prolonged parasite clearance and could be considered for implementation as molecular markers in the Greater Mekong Subregion, as a potential early signal of slow parasite clearance.

The confirmation of the apparent absence outside of Southeast Asia of common *pfk13* alleles associated with slow clearance is welcome. However, testing of a more practical and sensitive protocol than persistence of parasites on day 3 that can be a first signal of prolonged parasite clearance is urgently needed. Such an approach should also include vigilance for diminished artemisinin responses signaled by increasing prevalence of parasites with mutant alleles of *pfk13* or of other genetic changes that may also diminish parasite responses to artemisinins.

## Additional files

Additional file 1: Details of the systematic literature review. (PDF 213 kb)

Additional file 2: Supplementary Tables S1-S3. (XLSX 66 kb)

Additional file 3: Supplementary Figures S1- S9. (PDF 1168 kb)

## Abbreviations

ACTs: Artemisinin combination therapies
AL: Artemether/lumefantrine
AS + ACT: Artesunate + artemisinin combination therapy
AS: Artesunate
ASAQ: Artesunate/amodiaquine
ASMQ: Artesunate/mefloquine
DHAPIP: Dihydroartemisinin/piperaquine
DMSAP: Data Management and Statistical Analysis Plan
FN: False negative
FP: False positive
GMS: Greater Mekong Subregion
NS: Non-synonymous
OxTREC: Oxford Tropical Research Ethics Committee
PC_1/2_: Parasite clearance half-life
PCE: Parasite clearance estimator tool
RSA: Ring-stage survival assay
TRAC: Tracking resistance to artemisinin collaboration
WHO: World Health Organization
