# Supplementary material for: Association of mutations in the Plasmodium falciparum Kelch13 gene (Pf3D7_1343700) with parasite clearance rates after artemisinin-based treatments—a WWARN individual patient data meta-analysis
Source: BMC Med. 2019 Jan 17;17:1. doi: 10.1186/s12916-018-1207-3 (PMC6335805; doi:10.1186/s12916-018-1207-3)
Supplement: Supplementary file 1 — Details of the systematic literature review. (PDF 213 kb) [file 12916_2018_1207_MOESM1_ESM.pdf]

Table 1. Search strategy for the systematic review of literature

Database	Search Strategy
**Ovid MEDLINE(R) Epub Ahead of Print, In-Process & Other Non-Indexed Citations, Ovid MEDLINE(R) Daily, Ovid MEDLINE and Versions(R) <1946 to January 24, 2018>**	1 ((K13 or kelch) and (half life or parasite clearance)).mp. (71)<br>2 limit 1 to yr="2000 - 2017" (60)
**Pubmed**	(K13 or kelch) and (half life or parasite clearance)<br>Filters activated: Publication date from 2000/01/01 to 2017/12/31
**Embase <1974 to 2018 January 29>**	1 ((K13 or kelch) and (half life or parasite clearance)).mp. (134)<br>2 limit 1 to yr="2000 - 2017" (124)
**Web of Science Core Collection**	**TOPIC:** ((K13 OR kelch) AND (half life OR parasite clearance))<br>**Timespan:** All years. **Indexes:** SCI-EXPANDED, SSCI, A&HCI, CPCI-S, CPCI-SSH, BKCI-S, BKCI-SSH, ESCI, CCR-EXPANDED, IC.

Figure 1. Systematic Review Trial Profile

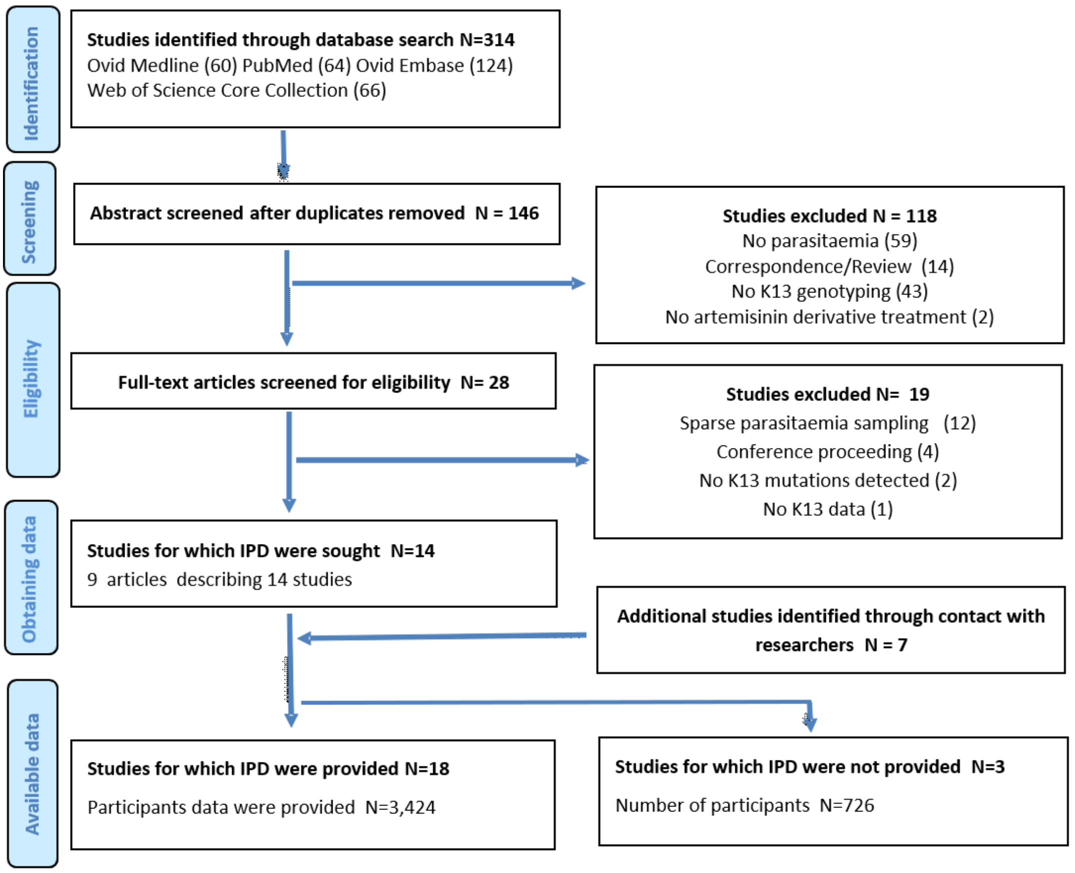

Assessment of study risk of bias.

Risk was classified as low L, moderate M, or high H using the following rules:

(a) molecular methods:

L = whole gene/propeller region genotyped; M = only specific mutations genotyped

(b) parasite sampling:

L = parasitaemia measured at least twice daily until clearance with 3 samples collected in the first 12h; M = parasitaemia measured twice daily

(c) $PC_{1/2}$ exclusions due to sparse data or unsatisfactory model fit (detailed criteria provided in Methods section and Figure 2):

L= < 20% of patients excluded; M = 20% -25% patients excluded; H = ≥25% patients excluded

Table 2. Risk of bias in individual studies.

Study ID	Risk of Bias		
	Molecular Methods	Parasite Sampling	$PC_{1/2}$ exclusion
1	L	L	L
2	L	L	L
3	L	L	M
4	L	L	L
5	L	L	L
6	L	L	L
7	L	M	H
8	M	M	L
9	L	L	L
10	L	L	H
11	L	L	H
12	L	L	L
13	L	L	M[1]
14	L	L	L
15	L	L	L
16	L	L	L
17	L	L	H
18	L	L	L

$PC_{1/2}$ = parasite clearance half- life

[1] the microscopy findings could not be confirmed due to the fact the slides had deteriorated
