# Supplementary material for: Association of mutations in the Plasmodium falciparum Kelch13 gene (Pf3D7_1343700) with parasite clearance rates after artemisinin-based treatments—a WWARN individual patient data meta-analysis
Source: BMC Med. 2019 Jan 17;17:1. doi: 10.1186/s12916-018-1207-3 (PMC6335805; doi:10.1186/s12916-018-1207-3)

Supplementary Figures

Supplementary Figure S1. **Distribution by study site in Africa of parasite clearance half-life in parasites from patients with no *pfk13* mutation**. The number in brackets shows number of isolates. The red line shows a half-life of 5.5 hours. The median is shown as a green circle, the red bar corresponds to the interquartile range, and the curve represents kernel estimate of the density function.

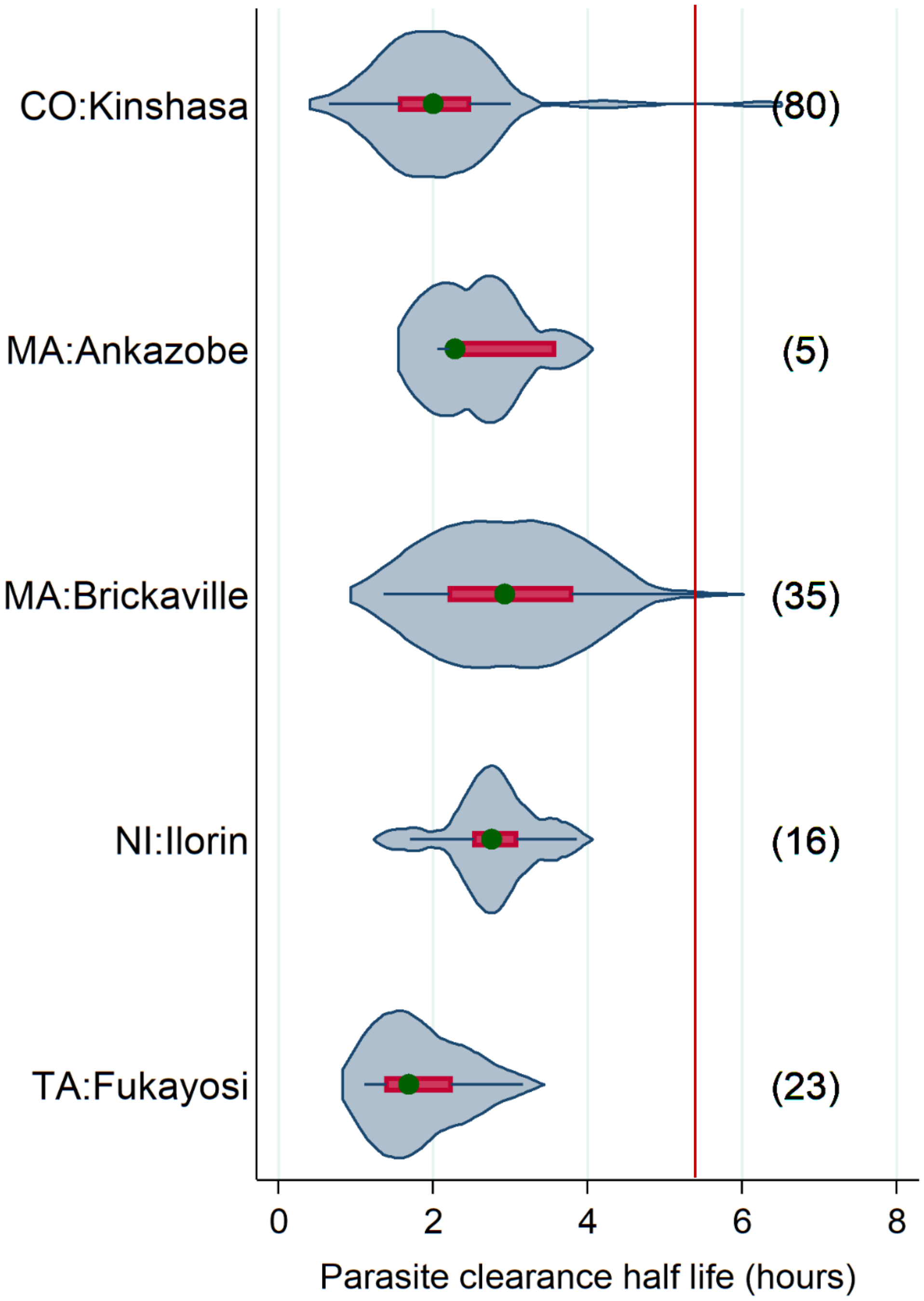

Supplementary Figure S2. Distribution by study site in Asia of parasite clearance half-life in parasites from patients with no *pfk13* mutation[1]. The number in brackets shows number of isolates. The red line shows a half-life of 5.5 hours. The median is shown as a green circle, the red bar corresponds to the interquartile range, and the curve represents kernel estimate of the density function.

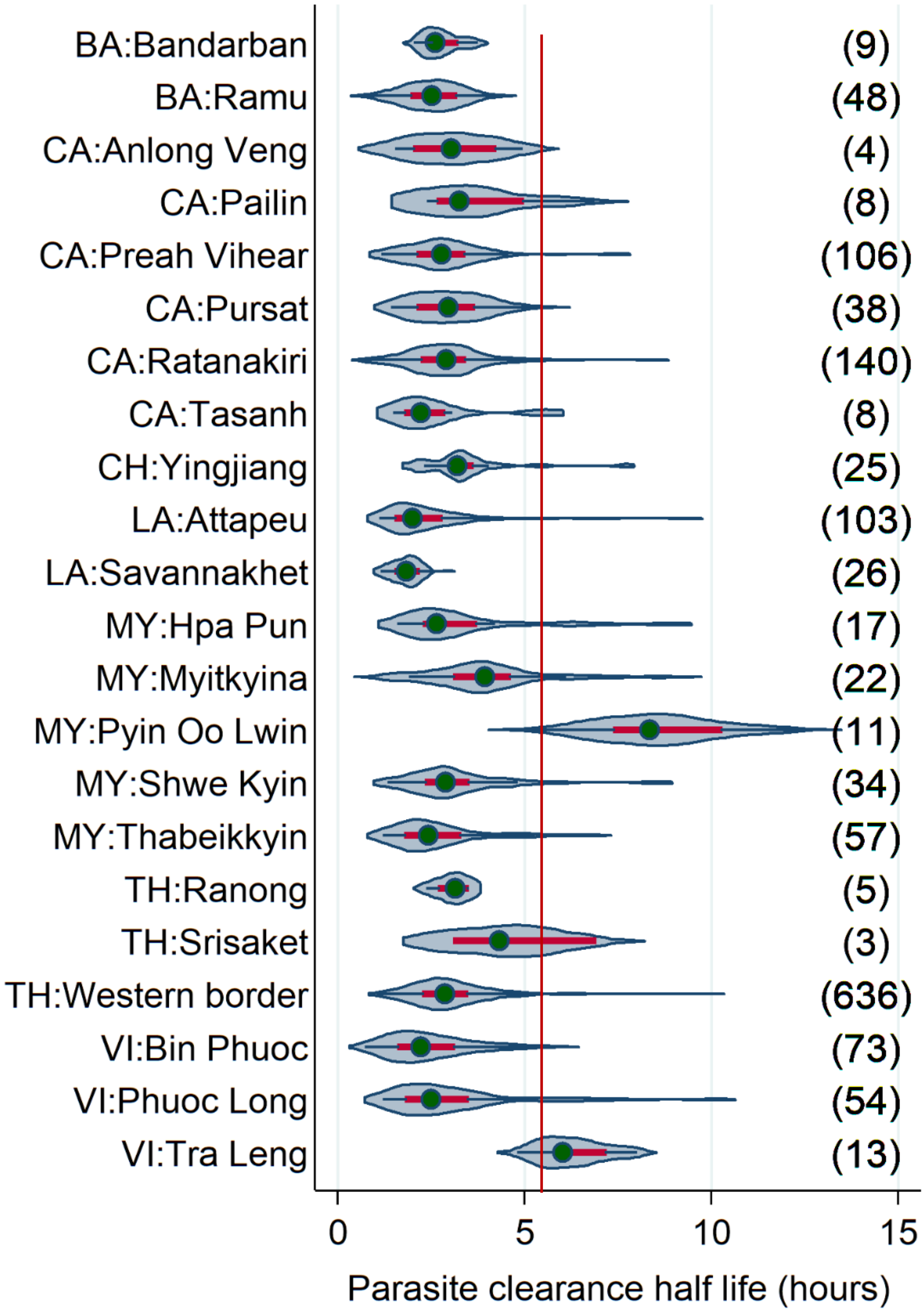

[1]The genotyping at Tra Leng, Vietnam was done to specifically identify the 4 pfk13 propeller mutant codons known at the time (Y493H, R539T, I543T, and C580Y) since the isolates presented here may carry mutations not yet identified. The high median half-life in isolates from Pyin Oo Lwin has been examined, but no explanation for the values was obtained. See explanation for the how these data were handled in bias section of the results and in the Table 3

Supplementary Figure S3. Distribution by study site of parasite clearance half-life in parasites with a *pf*k13 mutation at codon E252Q. The number in brackets shows the number of isolates. The red line shows a half-life of 5.5 hours. The median is shown as a green circle, the red bar corresponds to the interquartile range, and the curve represents kernel estimate of the density function.

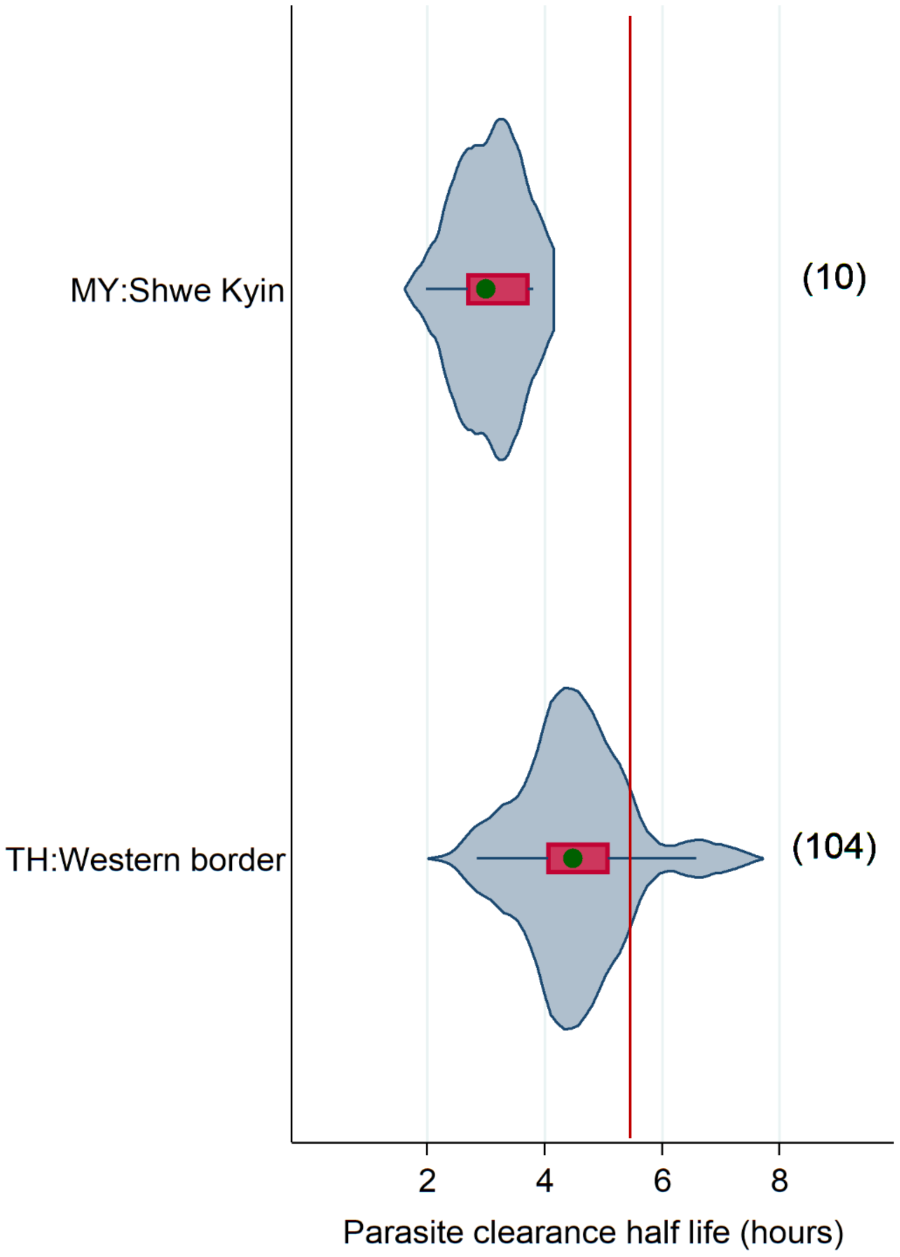

Supplementary Figure S4. Distribution by study site of parasite clearance half-life in parasites with *pf*k13 mutation at codon F446I. The number in brackets shows the number of isolates. The red line shows a half-life of 5.5 hours. The median is shown as a green circle, the red bar corresponds to the interquartile range, and the curve represents kernel estimate of the density.

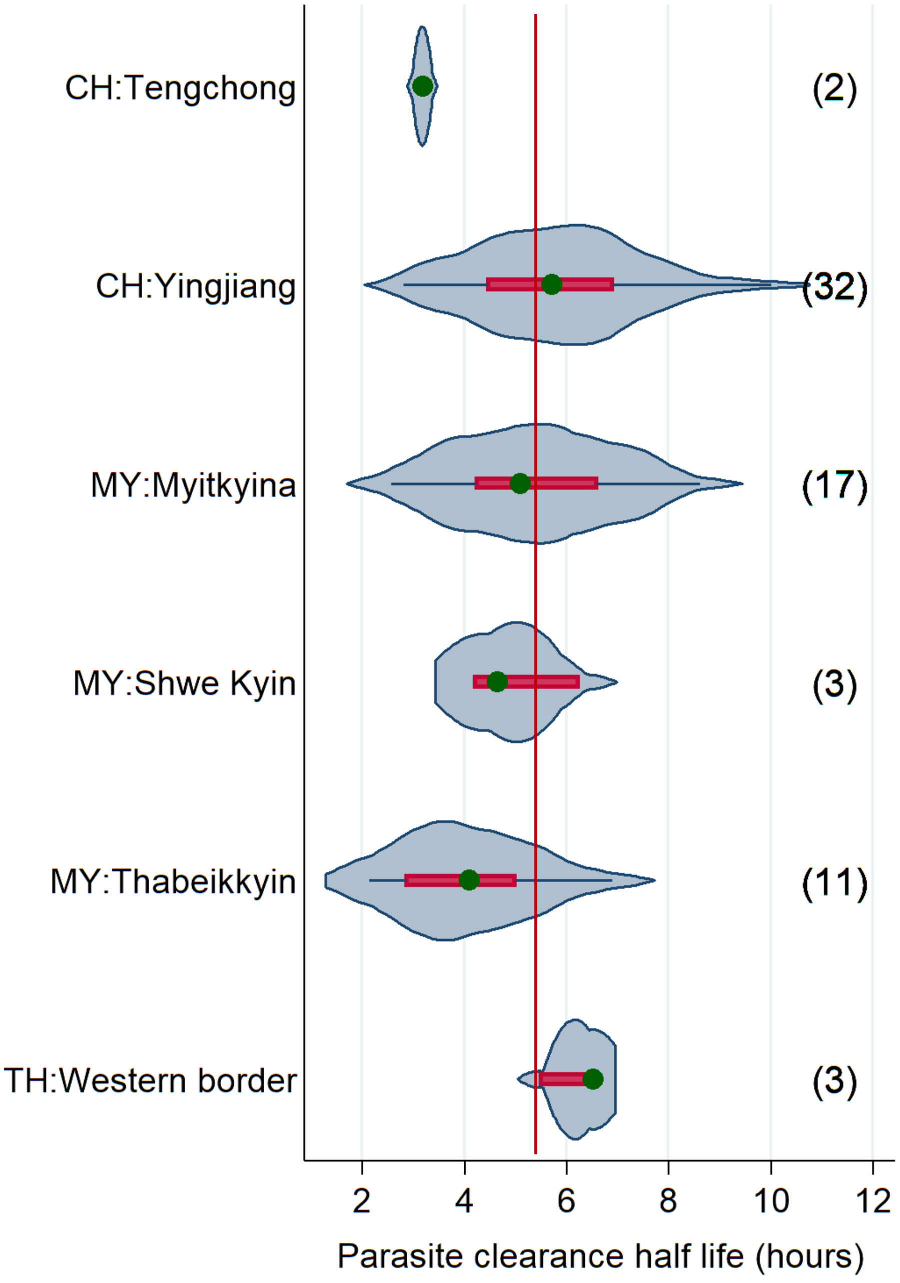

Supplementary Figure S5. Distribution of parasite clearance half-life in parasites with *pf*k13 mutation at codon Y493H by study site. The number in brackets shows the number of isolates. The red line shows a half-life of 5.5 hours. The median is shown as a green circle, the red bar corresponds to the interquartile range, and the curve represents kernel estimate of the density.

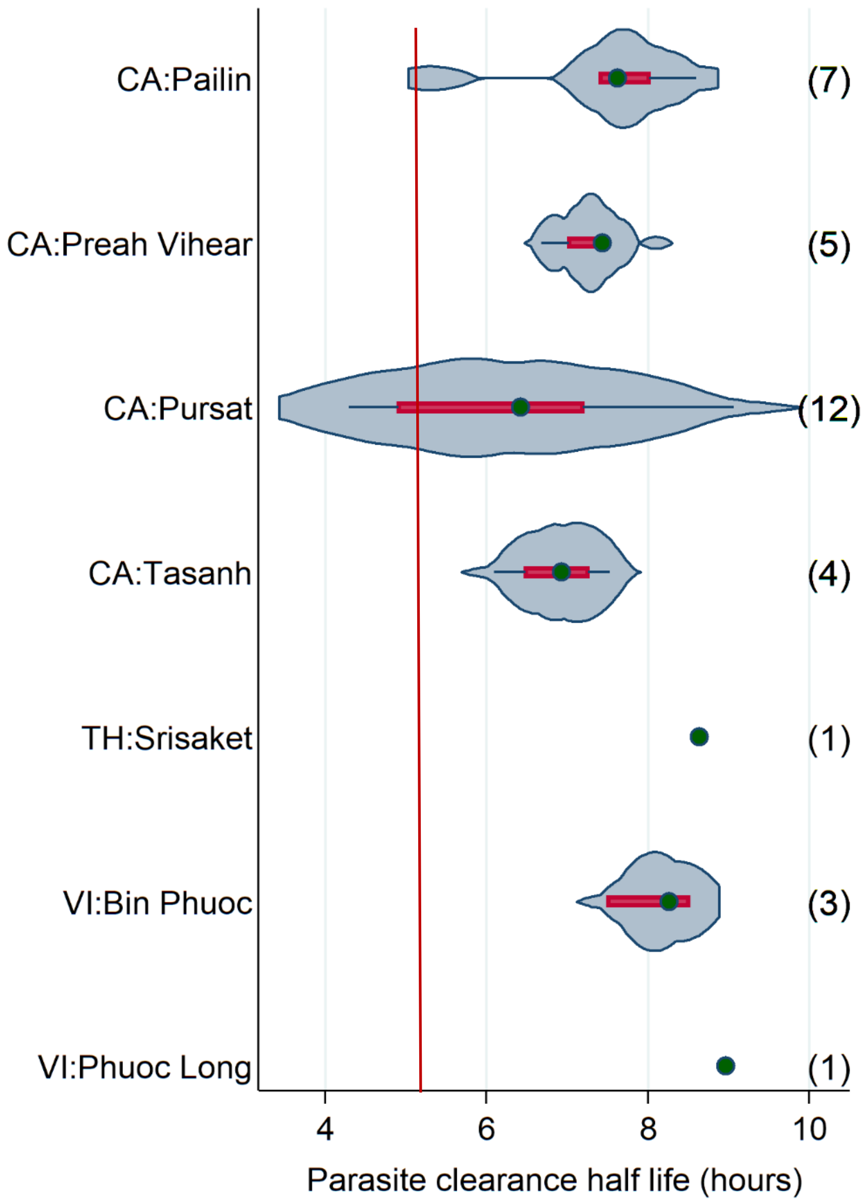

Supplementary Figure S6. Distribution by study site of parasite clearance half-life in parasites with *pf*k13 mutation at codon P574L[1]. The number in brackets shows the number of isolates. The red line shows a half-life of 5.5 hours. The median is shown as a green circle, the red bar corresponds to the interquartile range, and the curve represents kernel estimate of the density.

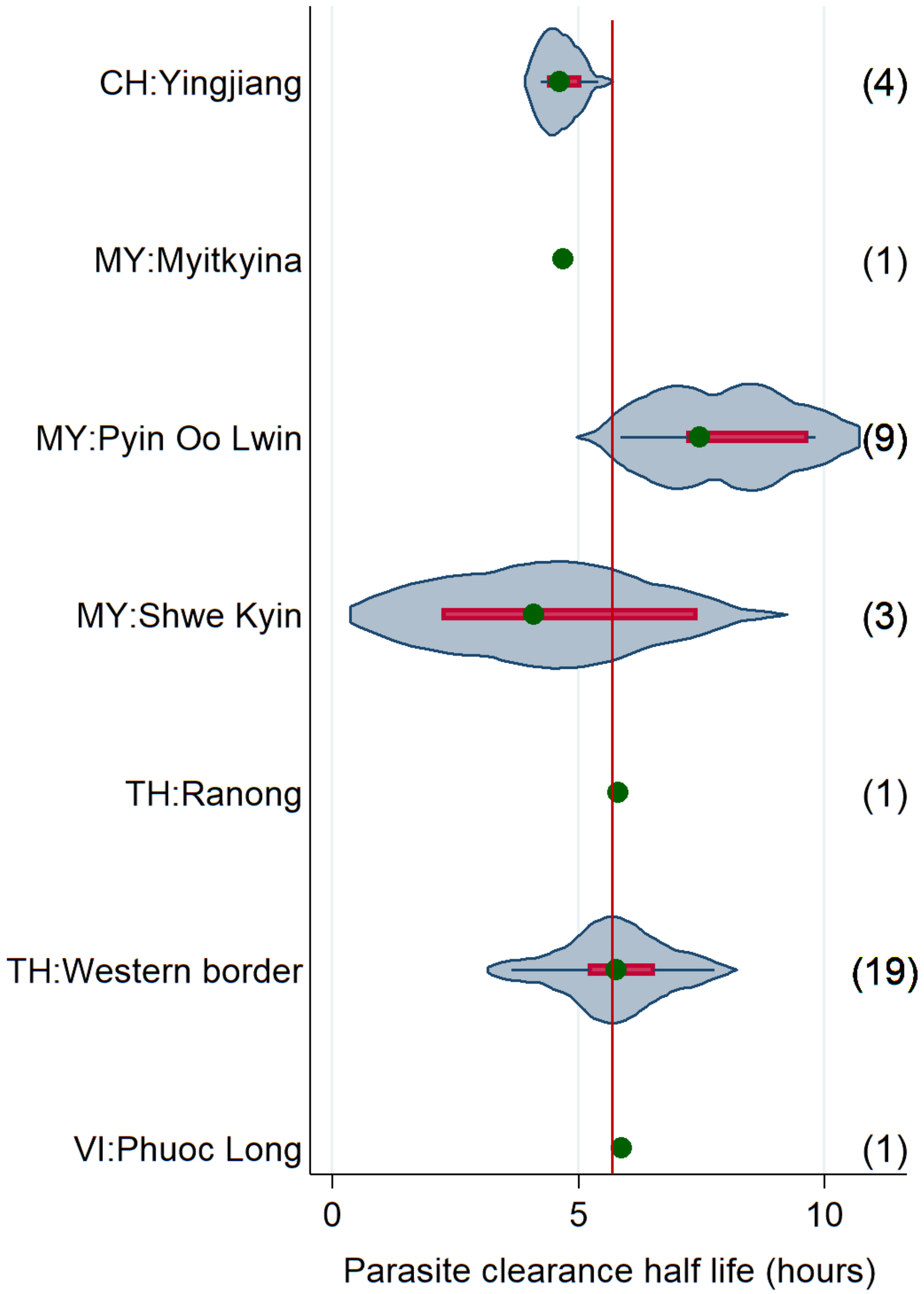

[1]The high median half-life in isolates from Pyin Oo Lwin has been examined, but no explanation for the values was obtained. See explanation for the how these data were handled in bias section of the results and in the Table 3 legend.

Supplementary Figure S7. Distribution by study site of parasite clearance half-life in parasites with *pf*k13 mutation at codon C580Y. The number in brackets shows the number of isolates. The red line shows a half-life of 5.5 hours. The median is shown as a green circle, the red bar corresponds to the interquartile range, and the curve represents kernel estimate of the density.

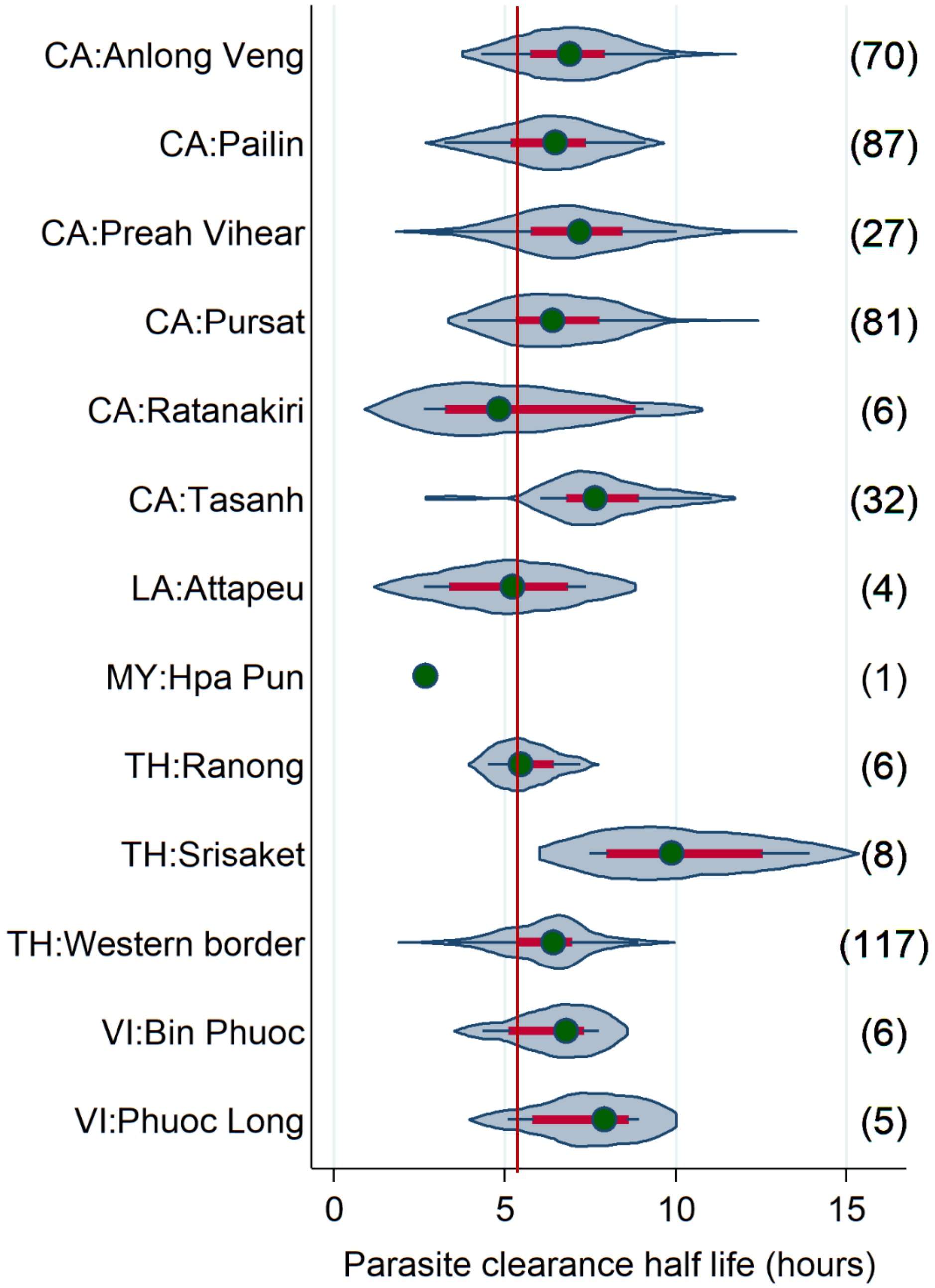

Supplementary Figure S8. Temporal trend in distribution of parasite clearance half-life isolates from the Western Border of Thailand that had parasites with C580Y *pf*k13 mutation. The temporal series of data shown are from study 4. Number in brackets shows number of patients. Median is shown as green circle, red bar corresponds to the interquartile range, curve represents kernel estimate of the density function.

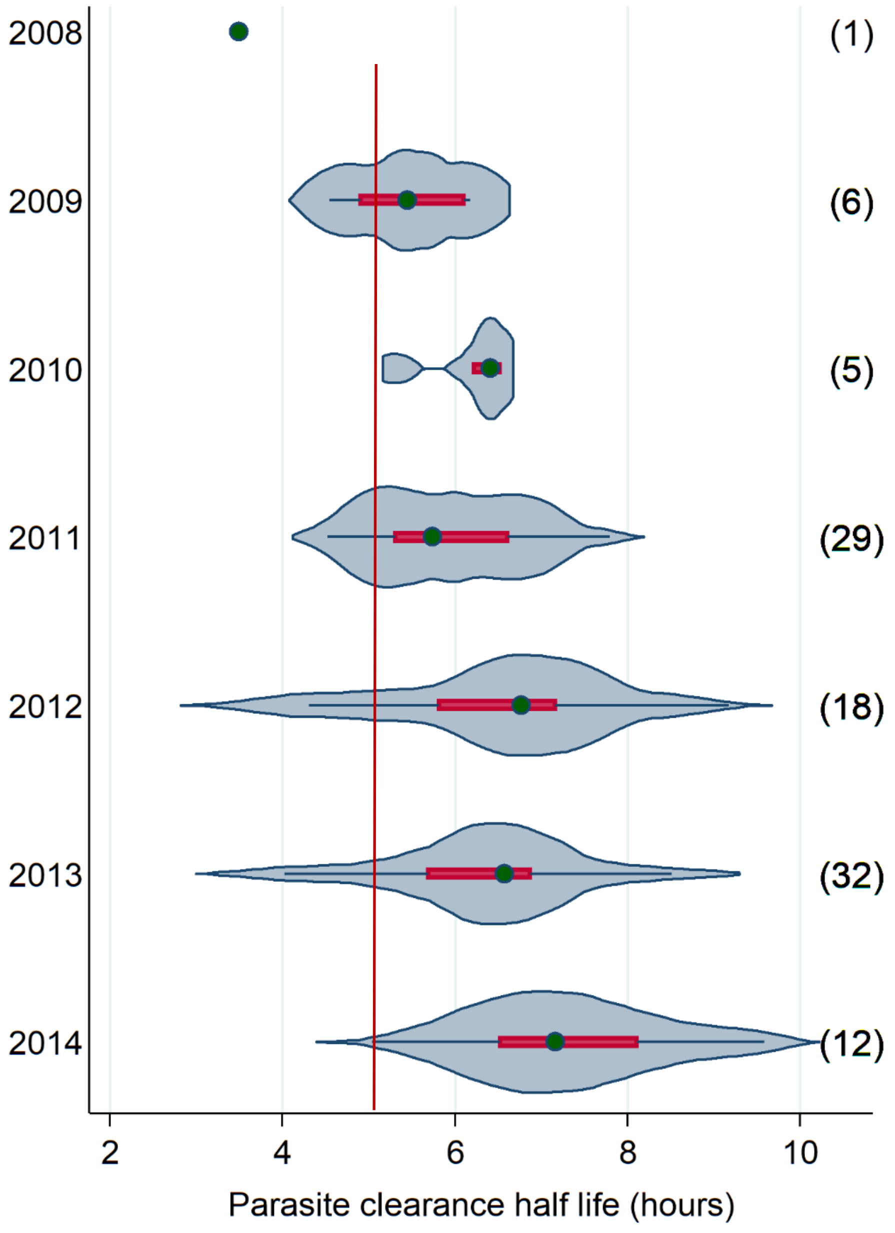

Supplementary Figure S9. Comparison of half-life and proportion of Day 3 positive isolates as a metric for slow clearance. Top panel (A) shows the proportion of patient isolates carrying each mutant codon with a half-life >5.5; Bottom panel (B) shows proportion of patient isolates that remained parasitemic by microscopy on day 3 after treatment.

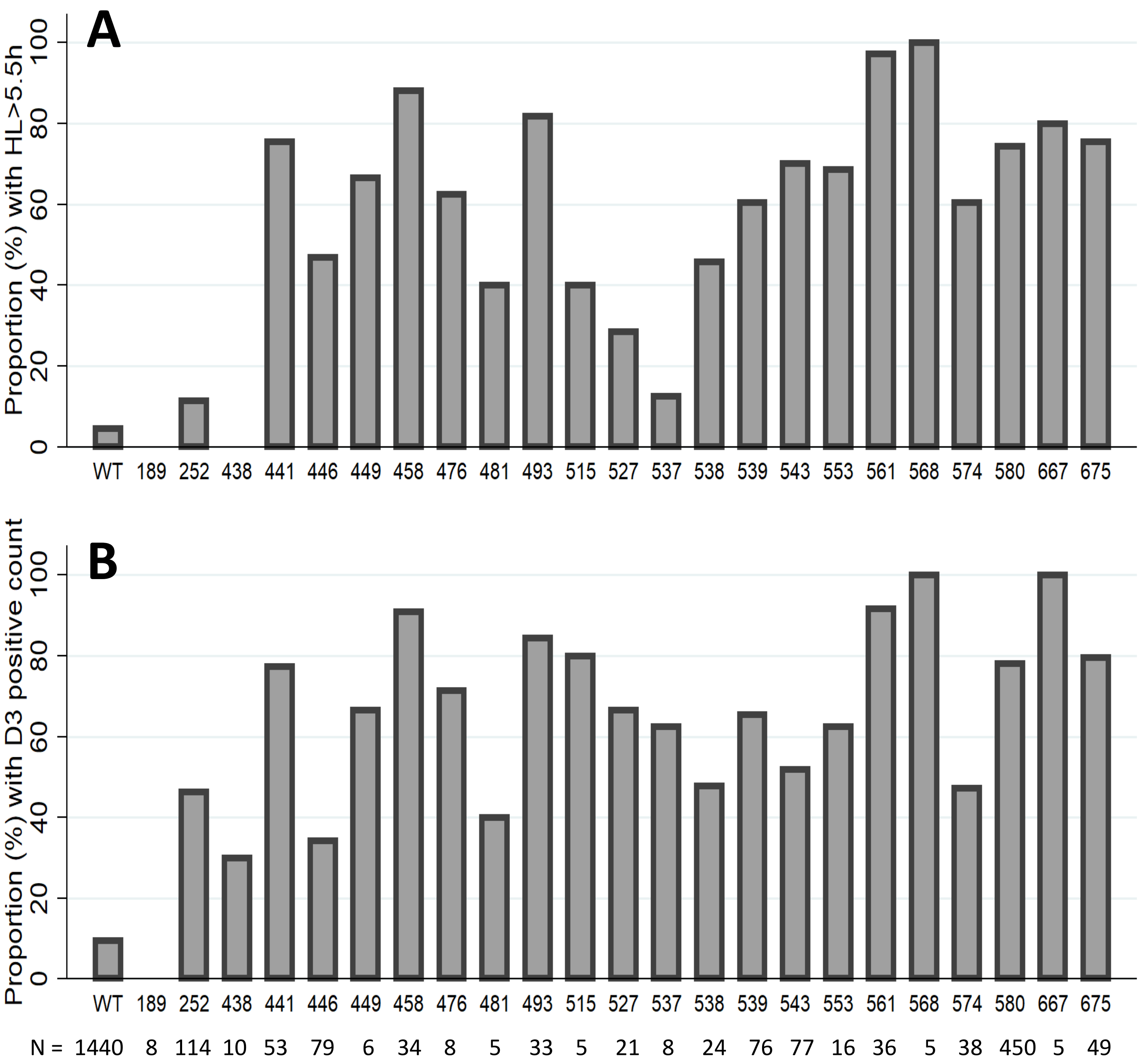

Supplement: Supplementary file 3 — Supplementary Figures S1- S9. (PDF 1168 kb) [file 12916_2018_1207_MOESM3_ESM.pdf]
